# Stem Cell Therapy for Inflammatory Diseases: Progress, Challenges, and Future Directions

**DOI:** 10.1002/mco2.70616

**Published:** 2026-02-02

**Authors:** Chen Wu, Zhi‐Ping Jin, Shu‐Qiang Weng, Ji‐Min Zhu, Ling Dong

**Affiliations:** ^1^ Department of Gastroenterology and Hepatology and Shanghai Institute of Liver Diseases Zhongshan Hospital Fudan University Shanghai China; ^2^ Department of Pharmacy, Zhongshan Hospital Fudan University Shanghai China

**Keywords:** hematopoietic stem cell, hematopoietic stem cell transplantation, induced pluripotent stem cell, inflammatory disease, inflammatory bowel disease, mesenchymal stem cell

## Abstract

Inflammatory diseases, encompassing conditions like inflammatory bowel disease and rheumatoid arthritis, present a significant clinical challenge with substantial treatment‐refractory patient populations despite biologic therapy advances. Stem cell therapeutics have emerged as a transformative approach, leveraging multifaceted regenerative mechanisms to address the complex pathophysiology of these conditions, which involves genetic, microbial, immunological, and epithelial dysregulation. This review focuses on comparing the clinical efficacy of contemporary stem cell strategies. We analyze outcomes across diverse cell sources, with a detailed examination of delivery methodologies. Our systematic analysis demonstrates superior efficacy with targeted delivery systems, particularly in managing localized inflammatory lesions (e.g., fistulas) and tissue restoration. Notably, minimally processed cellular interventions, such as autologous fat grafting and stromal vascular fraction therapy, show unexpected therapeutic promise. Critical translational barriers include suboptimal cell homing, limited engraftment persistence, and uncharacterized long‐term safety profiles. We propose strategic solutions through induced pluripotent stem cell platforms, precision genetic modifications, and advanced delivery technologies. By integrating mechanistic insights with robust clinical evidence, this review establishes an evidence‐based framework for optimizing stem cell therapeutics in inflammatory disease management. The analysis addresses fundamental scalability and safety considerations while identifying promising avenues for personalized regenerative medicine approaches in treatment‐refractory inflammatory conditions.

## Graphical abstract

Stem cells offer multifaceted therapeutic approaches for inflammatory diseases, each exhibiting distinct therapeutic properties. Hematopoietic stem cells, primarily through hematopoietic stem cell transplantation (HSCT), can effectively remodel the immune environment, showing particular promise in treating genetically linked cases. Mesenchymal stem cells (MSCs) demonstrate broad therapeutic potential through immunomodulation, tissue regeneration, intestinal microbiota regulation, and fistula repair. Tissue‐specific stem cells (TSCs) and induced pluripotent stem cells (iPSCs) currently serve primarily as research tools for understanding disease mechanisms and as platforms for drug screening. Furthermore, the potential of intestinal transplantation using TSC‐derived organoids represents a promising future therapeutic direction.

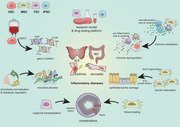

## 1 Introduction

Inflammatory diseases are characterized by tissue damage or dysfunction resulting from aberrant immune responses. This broad pathological concept encompasses both acute and chronic manifestations across numerous organ systems, representing a significant global health burden. These disorders encompass diverse pathological models across organ systems, broadly classified into major categories: (1) infectious inflammation (e.g., bacterial sepsis), (2) autoimmune diseases (e.g., systemic lupus erythematosus [SLE], rheumatoid arthritis [RA]), (3) allergic conditions (e.g., atopic dermatitis), (4) chronic noninfectious inflammation (e.g., hepatic/pulmonary fibrosis), and (5) other special conditions (e.g., graft‐versus‐host disease [GVHD]). Critically, while infectious inflammation often has effective antimicrobial therapies, many chronic immune‐mediated disorders like autoimmune diseases, allergic conditions and fibrotic disorders often lack curative treatments. Their pathogenesis is closely linked to genetic susceptibility, environmental factors, and disrupted immune homeostasis, independent of direct pathogenic stimulation. These conditions often exhibit a pattern of chronic remission and relapse, with symptoms including pain, swelling, tissue dysfunction, and systemic effects like fatigue. While the precise etiology of many inflammatory diseases remains elusive, current research suggests a complex interplay between genetic susceptibility and various environmental factors [1, 2, 3]. Despite ongoing investigations, the global incidence of numerous inflammatory diseases continues to rise [4, 5].

Conventional therapies for noninfectious inflammatory diseases (particularly autoimmune conditions), such as NSAIDs, corticosteroids, and immunosuppressants, often demonstrate limited long‐term efficacy. These treatments frequently necessitate prolonged and complex dosing regimens with regular adjustments, yet many patients remain refractory to these interventions [6]. Recent advancements in biologics, such as monoclonal antibodies (e.g., adalimumab and golimumab) and anti‐integrin molecules, have expanded treatment options for certain conditions [7, 8]. However, a significant proportion of patients fail to respond to current therapies, and many develop adverse reactions or lose responsiveness over time [9]. This therapeutic inadequacy is also pronounced for chronic fibrotic diseases, where limited pharmacological interventions may merely slow symptom progression without halting the underlying pathogenesis or reversing established damage [10, 11]. This therapeutic challenge underscores the urgent need for novel treatment modalities, among which stem cell therapy has emerged as a promising avenue of investigation [9].

Stem cells, defined by their self‐renewal capacity and potential to differentiate into multiple cell types, represent a unique class of multipotent progenitors. Their intrinsic immunomodulatory properties and tissue‐reparative functions position them as particularly valuable candidates for addressing the dual challenges of immune dysregulation and structural damage characteristic of refractory inflammatory diseases [12]. In these diseases, stem cell therapy offers a range of multifaceted potential benefits. These include promoting tissue healing and repair, modulating inflammatory responses, maintaining immune homeostasis, improving bacterial balance within the organ cavity, and potentially offering a path toward complete disease remission. Several excellent reviews have covered stem cell therapies for specific diseases, such as cardiovascular diseases [13, 14], liver diseases [15, 16], arthritis [17], and cancers [18].

Although the therapeutic role of specific stem cells in certain inflammatory diseases has been reviewed, strategies such as fat grafting receive less attention, despite growing evidence that stem cells mediate its effects. Therefore, this review instead takes a cell‐type‐centric approach. We will describe the different types of stem cell‐based therapies, including mesenchymal stem cells (MSCs) and induced pluripotent stem cells (iPSCs), and provide the major mechanisms of various stem cell therapies and their applications in major inflammatory diseases across different organ systems, especially those that are relatively lacking in effective treatments and have made certain progress in stem cell research. Notably, this review focuses specifically on nonmalignant inflammatory diseases, excluding malignancy‐associated inflammation. Challenges and limitations associated with translating stem cell techniques into clinical practice will also be discussed.

## 2 Mechanisms for Stem Cell Therapy in Inflammatory Diseases

**FIGURE 1:**
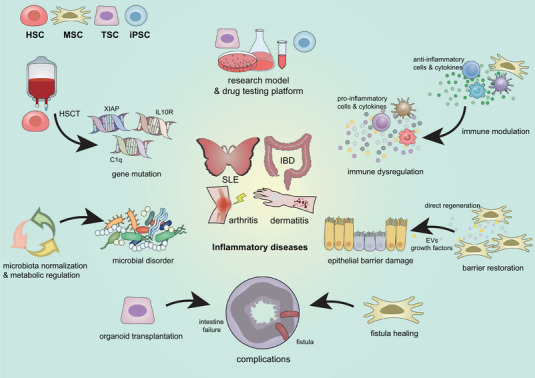
Mechanisms for stem cell therapy in inflammatory diseases. Stem cells offer multifaceted therapeutic approaches for inflammatory diseases, each exhibiting distinct therapeutic properties. Hematopoietic stem cells, primarily through hematopoietic stem cell transplantation (HSCT), can effectively remodel the immune environment, showing particular promise in treating genetically‐linked cases. Mesenchymal stem cells (MSCs) demonstrate broad therapeutic potential through immunomodulation, tissue regeneration, intestinal microbiota regulation, and tissue repair. Tissue‐specific stem cells (TSCs) and induced pluripotent stem cells (iPSCs) currently serve primarily as research tools for understanding disease mechanisms and as platforms for drug screening. Furthermore, the potential of transplantation using TSC‐derived organoids represents a promising future therapeutic direction.

In recent years, significant progress has been made in understanding stem cell therapy mechanisms for inflammatory diseases, targeting multiple pathophysiological factors that contribute to disease development and progression. Stem cell interventions target different inflammatory diseases through diverse mechanisms addressing several fundamental aspects: genetic susceptibility, immunological dysfunction, epithelial barrier disruption, and microbial dysregulation (Figure 1). Understanding these mechanisms provides crucial insights into the therapeutic potential of stem cell‐based treatments, highlighting promising directions for future research and clinical applications.

### 2.1 Genetic Factors

Interventions for genetic susceptibility remain limited; however, stem cell therapy represents one such approach. Hematopoietic stem cells (HSCs) have been extensively studied for decades, making them one of the most well‐characterized types of stem cells. While HSC transplantation (HSCT) was initially developed for treating leukemia, pioneering animal studies [19, 20] and serendipitous clinical observations [21] led to its application in immune disorders, including inflammatory bowel disease (IBD) [22] and SLE [23].

The rationale behind HSCT for inflammatory diseases is to eradicate dysfunctional bone marrow‐derived immune cells and reconstitute a healthy hematopoietic system. Notably, certain subtypes of inflammatory diseases exhibit strong associations with primary immunodeficiencies. Primary immunodeficiency diseases (PID) are a heterogeneous group of inherited disorders caused by genetic defects in immune system components, leading to increased susceptibility to infections, autoimmunity, and malignancy [24]. These subtypes often arise from monogenic mutations, such as those causing IL‐10R deficiency [25] and XIAP deficiency [26]. These genetic variants frequently result in severe symptom onset during infancy or early childhood. Many young patients with these conditions are refractory to conventional treatments and face high morbidity and mortality risks [27]. In such cases, allogeneic transplantation is highly recommended and may represent the only curative option [28, 29].

Beyond IBD, HSCT has demonstrated significant efficacy in SLE. While most SLE cases involve polygenic risk factors, the study of monogenic SLE subtypes has provided critical mechanistic insights. These studies have particularly revealed that defects in nucleic acid clearance can trigger aberrant type I interferon responses and B‐cell autoreactivity [30]. For patients with SLE caused by C1q deficiency, HSCT aims to eliminate autoreactive immune memory by ablating pathogenic cell populations (like abnormal C1q‐producing monocytes) and then restore C1q levels through the transplantation [31].

### 2.2 Microbial Factors

Treatment of colitis with MSCs has demonstrated the ability to restore microbiota composition and function in both dextran sulfate sodium (DSS)‐ and 2,4,6‐trinitrobenzenesulfonic acid‐induced colitis models, effectively reducing inflammation to normal levels [32, 33]. Researchers suggest that the potential mechanism underlying this effect may be related to the interaction between gut microbiota and host metabolism. Recent mechanistic investigations using human umbilical cord‐derived MSCs in colitis mice revealed specific normalization of Bacteroidetes/Firmicutes ratios [32]. Additionally, several metabolic pathways, including sulfur and riboflavin metabolism, amino acid biosynthesis, lysine biosynthesis, sphingolipid metabolism, and secondary bile acid biosynthesis, can be modulated by MSC treatment [32]. Notably, several studies have specifically highlighted the regulatory effects of extracellular vesicles (EVs), particularly stem cell‐derived exosomes, on the gut microbial environment and their partial therapeutic contributions [34]. The cell‐free nature of EVs may provide enhanced access to the intestinal lumen, allowing direct modulation of microbial metabolic activities.

Notably, current understanding of MSC–microbiome interactions remains preliminary, primarily confined to IBD models. However, emerging evidence suggests broader implications as microbial dysregulation is increasingly implicated in diverse inflammatory diseases [35, 36]. More notably, nucleic acids of certain microbiota have been detected in some previously considered sterile environments, such as the joint cavities of RA patients [37]. This raises a critical hypothesis as to whether the therapeutic effects of intra‐articular MSC administration in RA be partially mediated through modulation of local microbial communities, analogous to mechanisms observed in IBD. However, direct evidence linking MSC therapy to synovial microbiome remodeling remains absent. This represents a significant knowledge gap warranting further investigation.

### 2.3 Immunological Factors

**FIGURE 2:**
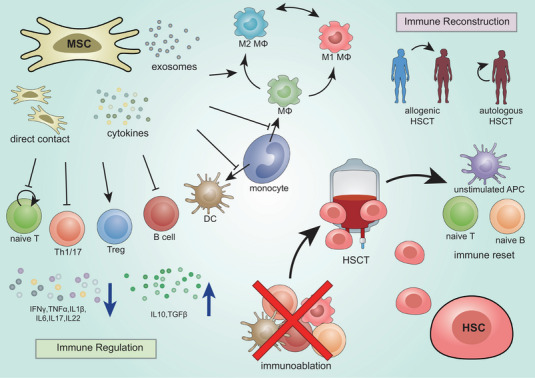
Immunomodulatory mechanisms of HSCs and MSCs. Mesenchymal stem cells (MSCs) exhibit broad immunomodulatory effects through multiple mechanisms, including direct cell–cell contact and paracrine signaling via secreted cytokines and exosomes. These interactions promote the transition from proinflammatory to anti‐inflammatory immune cell phenotypes. Conversely, hematopoietic stem cell transplantation (HSCT) employs a two‐step therapeutic approach: first, immunoablation eliminates dysregulated immune cells, providing initial therapeutic benefit; second, transplanted hematopoietic stem cells (HSCs) reconstitute the immune system, establishing a restored immunological tolerance.

Stem cell therapy shows a broad spectrum of immunomodulatory capabilities (Figure 2). HSCs have the potential to renew an abnormal immune system. The infusion of HSCs can effectively “reset” a patient's immune system to a naive and tolerant immune state through multiple mechanisms, including thymic activation‐mediated acquisition of T‐cell receptor diversity [38], upregulation of regulatory T cells [39], and regeneration of the naive B‐cell compartment [40]. MSCs, on the other hand, have been shown to exert potent immunomodulatory effects through various pathways, including cell‐to‐cell contact with both innate and adaptive immune cells, the secretion of anti‐inflammatory molecules, and the regulation of the immune microenvironment [41]. Preclinical evidence demonstrates the multilayered immunoregulatory abilities of MSCs, operating at both cellular and cytokine levels. At the cellular level, MSCs downregulate Th1‐mediated inflammation [42], restore Treg/Th17/Th1 equilibrium [43, 44, 45], enhance IL‐10‐producing Breg expression [44], promote M1‐to‐M2 macrophage polarization [46, 47], and modulate dendritic cell activity and quantity [48, 49]. At the cytokine level, MSCs secrete immunosuppressive factors, including IL‐10 [50] and TSG‐6 [46], collectively orchestrating immune responses. These diverse mechanisms contribute to the therapeutic potential of stem cells in modulating immune responses.

### 2.4 Tissue Repair Factors

Initially, the prorepair capacity of stem cells was mainly attributed to their direct differentiation [51]. However, it is now well established that the regenerative potential of stem cells extends far beyond their “stemness”. For IBD, early animal experiments suggested that the therapeutic effects of MSCs on colitis may be mediated through multiple mechanisms, including the delivery of growth factors to injured regions [51], induction of angiogenesis [52], and reassembly of junction proteins [53]. These mechanisms collectively contribute to maintaining epithelial cell integrity and promoting tissue regeneration. A recent study has further elucidated and expanded upon the role of tumor necrosis factor‐inducible gene 6 protein (TSG‐6) in this process. It has demonstrated that MSCs promote epithelial cell proliferation via TSG‐6 in an Akt‐dependent manner through the hyaluronan–CD44 interaction, thereby accelerating mucosal healing [54]. Moreover, MSCs have been found to ameliorate colitis in mice by increasing circulating levels of insulin‐like growth factor 1 (IGF‐1). This increased IGF‐1 maintains epithelial cell integrity through the IGF1R–PI3K–AKT pathway, contributing to cellular repair and regeneration [55].

While in RA, MSC‐based therapies mediate tissue repair primarily through noncoding RNA [56]. Exosomal miR‐150‐5p directly suppresses matrix metalloproteinase 14 (MMP14) and vascular endothelial growth factor (VEGF) expression in fibroblast‐like synoviocytes, inhibiting cell migration/invasion and pathological angiogenesis, thereby ameliorating joint destruction in collagen‐induced arthritis models [57]. Concurrently, exosomal circEDIL3 acts as a molecular sponge for miR‐485‐3p, leading to upregulation of protein inhibitor of activated STAT‐3 (PIAS3), known as a negative regulator of STAT3 signaling, which ultimately attenuates VEGF production and abnormal pannus development [58]. These RNA‐directed mechanisms synergistically target synovial hyperplasia and neovascularization, establishing MSC‐exosomes as potent regulators of joint microenvironment homeostasis and joint repair in RA.

The therapeutic effects of stem cell interventions in inflammatory diseases are inherently multifaceted and interconnected, presenting both opportunities and challenges for clinical translation. This mechanistic complexity theoretically confers advantages over conventional single‐target therapies by simultaneously addressing multiple pathogenic pathways through a single therapeutic modality; however, it considerably complicates efforts to elucidate the precise therapeutic mechanisms underlying observed clinical benefits. The intricate crosstalk between these mechanisms (e.g., MSC‐mediated microbiome restoration enhancing tissue repair via microbial metabolite signaling) creates complex biological responses that resist reductionist analysis. Furthermore, current mechanistic understanding remains heavily skewed toward MSCs, which constitute approximately 80% of preclinical studies in this domain [32, 42, 43, 44, 45, 46, 47, 48, 49, 50, 51, 53, 54, 55, 59, 60, 61, 62, 63, 64, 65, 66, 67, 68, 69, 70, 71, 72, 73, 74, 75, 76, 77, 78, 79] (Table 1). The field's disproportionate focus on MSCs is due to their accessibility, low immunogenicity, and demonstrated efficacy in fistulizing disease, yet it has inadvertently marginalized research on equally promising cell types. Consequently, the therapeutic mechanisms of alternative stem cell populations remain inadequately characterized. HSCs are primarily studied through clinical outcomes rather than mechanistic pathways, despite their curative potential in refractory autoimmune conditions. iPSCs lack standardized differentiation protocols for mechanistic interrogation, despite their capacity to model patient‐specific pathologies. Tissue‐specific stem cells (TSCs, e.g., intestinal/dermal/pulmonary stem cells) suffer from technical limitations in isolation and expansion. These knowledge gaps fundamentally constrain our ability to optimize cell‐type selection, predict treatment responses, and engineer next‐generation therapies.

**TABLE 1:** Preclinical evidence of animal experiments of stem cell therapy in inflammatory diseases.

Year	Disease model	Cell source	Administration method	Mechanism	References
2008	Colitis	Rat bone marrow	Submucosal injection	Differentiate into colonic interstitial cells and provide VEGF and TGF‐beta1	[51]
2009	Colitis	Rat bone marrow	Intravenous injection	Maintain epithelial barrier function by reassembling tight junction proteins	[53]
2009	Colitis	Human adipose	Intraperitoneal injection	Downregulate Th1‐driven inflammatory responses	[42]
2013	Colitis	Human umbilical cord	Intraperitoneal injection	Balance Treg/Th17	[43]
2015	Colitis	Human umbilical cord	Intraperitoneal injection	Reduce intestinal permeability and upregulate the expression of tight junction proteins	[59]
2016	Colitis	Human umbilical cord	Intraperitoneal injection	Boost IL‐10‐producing CD5+ Bregs and balance Treg/Th17/Th1	[44]
2018	Colitis	Canine adipose	Intraperitoneal injection	Secrete TSG‐6 to switch M1 macrophages to M2	[46]
2018	Colitis	Mouse adipose	Intraperitoneal injection	Increase regulatory dendritic cells	[48]
2018	Colitis	Mouse bone marrow	Intravenous injection	Suppress dendritic cells	[49]
2019	Colitis	Human adipose	Intravenous injection	Upregulate M2 macrophages and regulatory T cells	[47]
2019	Colitis	Human gingiva	Intravenous injection	Modulate IL‐10 signal	[50]
2019	Colitis	iPSC	Intraperitoneal injection	Promote epithelial cell proliferation via TSG‐6	[54]
2020	Colitis	Rat adipose	Intravenous injection	Boost intestinal epithelial cell regeneration, regulate Wnt signaling, and disturb T cell immunity	[60]
2020	Colitis	Human umbilical cord	Intraperitoneal injection	Increase regulatory T cells	[45]
2020	Colitis	Human embryonic stem cell	Intravenous injection	Elevate circulating IGF‐1 to maintain the integrity of epithelial cells	[55]
2022	Colitis	Human umbilical cord	Intraperitoneal injection	Normalize gut microbiota	[32]
2016	Psoriasis	Human umbilical cord	Intradermal injection	Inhibit proinflammatory cytokines and chemokines	[61]
2018	Psoriasis	Human tonsil	Subcutaneous injection	Prevent Th17‐mediated response via PD‐1/PD‐L1 pathway regulation	[62]
2022	Psoriasis	Human umbilical cord	Subcutaneous injection	Suppress IL‐17‐producing γδ T cells	[63]
2015	Atopic dermatitis	Human umbilical cord	Subcutaneous injection	Reduce mast cell degranulation	[64]
2017	Atopic dermatitis	Human adipose	Intravenous injection	Regulate B lymphocyte maturation	[65]
2019	Atopic dermatitis	Human umbilical cord	Subcutaneous injection	Reduce T cell responses	[66]
2020	Atopic dermatitis	Human umbilical cord	Subcutaneous injection	Inhibit secretion of TNF‐α and IgE	[67]
2022	Atopic dermatitis	Human umbilical cord	Subcutaneous injection	Regulate inflammatory responses of keratinocytes/Th2 cells/mast cells	[68]
2018	GVHD	Human placenta	Intravenous injection	Modulate Th17/Tr1 balance via expression of PD‐L2	[69]
2020	GVHD	Human placenta	Intravenous injection	Promote the generation of GSH and GST in PD‐1+ T cells	[70]
2023	GVHD	Human placenta	Intravenous injection	Modulate IFN‐γ and IL‐10 secretion by CD4+ T cells	[71]
2024	GVHD	Human placenta	Intravenous injection	Regulate TNF‐α and IL‐10 level in Th1 Cells	[72]
2024	GVHD	Human placenta	Intravenous injection	Promote the formation of Tregs	[73]
2025	GVHD	Human placenta	Intravenous injection	Regulate antioxidant capacity	[74]
2011	Arthritis	Human adipose	Intravenous injection	Inhibit various inflammatory mediators	[75]
2015	Arthritis	Human adipose	Intraperitoneal injection	Induce expression of FCGIIB receptors	[76]
2022	Lupus	Mouse bone marrow	Intravenous injection	Promote efferocytosis and recruitment of IL‐17+ regulatory T cell	[77]
2022	Lupus	Human umbilical cord	Intravenous injection	Induce M2 macrophage polarization and regulatory T cell expansion	[78]
2023	Lupus	Human umbilical cord	Intravenous injection	Inhibit the proliferation and differentiation of B cells	[79]

## 3 Stem Cell Types and Their Applications

Stem cell‐based therapies represent a promising avenue for treating inflammatory diseases, with multiple cell types showing therapeutic potential. The field encompasses HSCs, MSCs, TSCs, and iPSCs, each offering distinct advantages. These cells can be administered either systemically to modulate immune responses or locally to target specific inflammatory lesions, such as fistulas in IBD and joint synovitis in RA. Understanding their functional differences can optimize therapeutic strategies across diverse disease contexts.

### 3.1 General Cell Types

Several types of stem cells have shown promise in inflammatory diseases research and treatment: First, HSCs remain the only potential “curative” option for severe monogenic related disorders. Significant efforts are underway to enhance the safety and efficacy of HSC transplantation procedures. Second, MSCs are of particular interest due to their low immunogenicity and therapeutic efficacy in refractory conditions, such as fistulizing Crohn's disease (CD; FCD). Current research priorities focus on elucidating the therapeutic mechanisms of MSCs, which may inform the refinement of minimal therapeutic units. Third, TSC‐related technologies provide a novel and promising platform for disease modeling and drug testing in barrier repair research. The potential for organoid/ISC (intestinal stem cell) transplantation is substantial. Fourth, iPSCs offer a stable, artificial source of various stem cell types, which is particularly valuable in scenarios where direct patient samples are unavailable.

While different stem cell types may lead to distinct research directions, their therapeutic mechanisms in inflammatory diseases often overlap. A comprehensive approach to studying these cell types will facilitate a comparison of their advantages and limitations, potentially leading to integrated applications in the future.

### 3.2 Routes of Administration

Clinical research on stem cell‐based therapies has evolved along two primary tracks, reflecting the diverse mechanisms by which stem cells exert their therapeutic effects in inflammatory conditions. Broadly, systemic administration is typically employed for diseases characterized by systemic or widespread inflammation and immune dysregulation, such as SLE and refractory IBD. Conversely, local administration is favored for targeting localized tissue inflammation and damage, including inflammatory fistulae, localized skin conditions, and confined joint pathologies. Consequently, these distinct therapeutic strategies involve not only the application of different stem cell types but also necessitate separate evaluation frameworks and have distinct developmental trajectories, as detailed below.

#### 3.2.1 Systemically Administered Stem Cells

Systemic delivery remains a cornerstone for many stem cell therapies targeting inflammatory diseases, and it is the primary route of administration for most HSC therapies [80, 81, 82, 83, 84, 85, 86, 87, 88, 89, 90, 91, 92, 93, 94, 95, 96, 97, 98, 99, 100, 101, 102, 103, 104, 105, 106, 107, 108, 109, 110, 111, 112, 113, 114, 115, 116, 117, 118, 119, 120, 121, 122, 123, 124] (Table 2). The procedure involves eradicating the patient's overreactive immune system through a conditioning regimen, followed by the introduction of HSCs to restore immune tolerance. Given that the vast majority of HSC applications employ systemic delivery, with primary distinctions manifesting in cell sources, these aspects will be comprehensively addressed in the “source of stem cell” section below.

**TABLE 2:** Major clinical trials of systemic MSC infusion in inflammatory diseases.

Year	Indication	Phases	Patients	MSC origin	Dosage	Follow up (month)	Efficacy	Adverse event	NCT No. (status)	References
2006	GVHD	NA	8	Allogeneic BMSC	0.7–9 × 10^6^ cells/kg, 1 time	>40	75% overall proven response	No relevant AE	NA	[125]
2007	GVHD	NA	6	Allogeneic ADSC	1 × 10^6^ cells/kg, 1–2 times	40 (18–90)	83.3% overall proven response	No relevant AE	NA	[80]
2008	GVHD	II	55	Allogeneic BMSC	0.4–9 × 10^6^ cells/kg, 1–2 times	>24	54.5% complete response; 16.4% improvement	No relevant AE	NA	[81]
2011	GVHD	I/II	18	Allogeneic BMSC	1–4 × 10^6^ cells/kg, 1–2 times	NA	10% complete response, 60% partial response (acute GVHD); 12.5% complete response, 37.5% partial response (chronic GVHD)	No SAE	NCT00447460 (unknown status)	[82]
2011	GVHD	I	19	Allogeneic BMSC	1–4 × 10^6^ cells/kg, 2 (median) times	NA	58.3% complete response, 33.3% partial response (acute GVHD); 28.6% complete response, 42.9% partial response (chronic GVHD)	Mild abnormalities in taste	NA	[83]
2013	GVHD	NA	50	Allogeneic BMSC	0.3–3.1 × 10^6^ cells/kg, 1–4 times	>43	66% initial response; 34% complete resolution	No relevant AE	NA	[84]
2013	GVHD	I	40	Allogeneic BMSC	1.5 × 10^6^ cells/kg, 3 (median) times	4	67.5% complete response, 27.5% partial response	No relevant AE	NCT01764100 (unknown status)	[85]
2016	GVHD	II/III	25	Allogeneic BMSC	7.5–10 × 10^7^ cells, 4–8 times	4–52	24% complete response, 36% partial response	No relevant AE	NA	[86]
2017	GVHD	NA	46	Allogeneic BMSC	6.81 × 10^6^/kg cells/kg (accumulative)	60	50% clinical improvement; 13% complete response, 61% partial response	1 nausea; 1 blurred vision	NA	[87]
2018	GVHD	NA	33	Allogeneic BMSC	1–4 × 10^6^ cells/kg, 2 times	3	21.6% complete response, 30% partial response	3 SAE (ARDS, TTP, heart failure)	NCT00603330 (recruiting)	[88]
2018	GVHD	NA	69	Allogeneic BMSC	1–2 × 10^6^ cells/kg, 1–4 times	8.1	61% complete response, 25% partial response	1 nausea; 1 headache	NA	[89]
2020	GVHD	III	260	Allogeneic UCSC	2 × 10^6^ cells/kg, 8 times	6	35% durable complete response (MSC group), 30% in control, *p* = 0.42	No relevant AE	NCT00366145 (completed)	[90]
2020	GVHD	I	15	iPSCs	1/2 × 10^6^ cells/kg, 2 times	3.3	86.7% overall response, 53.3% complete response, and 86.7% overall survival	No relevant AE	NCT02923375 (completed)	[91]
2020	GVHD	I	10	Allogeneic WJSC	2/10 × 10^6^ cells/kg, 2 times	6	70% overall response, 40% complete response and 30% partial response	No relevant AE	NCT03158896 (recruiting)	[92]
2024	GVHD	III	130	Allogeneic UCSC	1 × 10^6^ cells/kg, 4/8 times	13	83.1% complete response (MSC group), 55.4% complete response (control), *p* = 0.001	No relevant AE	NCT04738981 (completed)	[93]
2024	GVHD	III	78	Allogeneic UCSC	1 × 10^6^ cells/kg, 8/16 times	24	Overall response: 60% (MSC group), 50% (control), *p* = 0.375	No relevant AE	NCT00366145 (completed)	[94]
2025	GVHD	NA	57	Allogeneic BMSC	1 × 10^6^ cells/kg, 1–6 times	<120	39% (days 14)/42% (days 28) overall response rate	NA	NA	[95]
2025	GVHD	Ib/IIa	25	Allogeneic UCSC	0.5/1/2 × 10^6^ cells/kg,6 times	4	40% complete response, 40% partial response	No relevant AE	NA	[96]
2010	CD	I	10	Autologous BMSC	1–2 × 10^6^ cells/kg, 2 times	1.5	30% clinical response; 20% endoscopic improvement; 30% worsening	1 AE (mild allergy); 0 SAE	NA	[97]
2010	UC	NA	44	Allogeneic BMSC	NA	24	NA	NA	NA	[98]
2011	CD and UC	NA	7	Allogeneic BMSC/UCSC	1 × 10^6^ cells/kg, 1 time	19 (6–32)	71% clinical remission; 43% endoscopic improvement	3 AE (face hot, insomnia, low fever)	NA	[99]
2014	CD	II	16	Allogeneic BMSC	2 × 10^6^ cells/kg, 4 times	1.5	50% clinical remission; 44% endoscopic improvement	16 related AE (dysgeusia); 1 SAE	NCT01090817 (completed)	[100]
2016	UC	I/II	70	Allogeneic UCSC	0.5–1.5 × 10^6^ cells/kg, 1 time	24	UCSC group: 85.3% clinical response/remission; control: 15.7%	No evident AE	NCT01221428 (unknown status)	[101]
2016	CD	I	12	Allogeneic BMSC	2/5/10 × 10^6^ cells/kg, 1 time	3	42% clinical response	2 related SAEs (appendicitis, Clostridium difficile colitis)	NA	[102]
2018	CD	I/II	13	Allogeneic BMSC	1.5–2.0 × 10^6^ cells/kg, 2 times	3	15% clinical remission; 15% worsening	1 AE (mild upper respiratory tract infection); 0 SAE	NCT01540292 (terminated)	[103]
2018	CD	NA	82	Allogeneic UCSC	1 × 10^6^ cells/kg, 4 times	12	0 clinical remission; 15% fistula improvement (UCSC group)	13 AE (fever, upper respiratory tract infection); 0 SAE	NCT02445547 (completed)	[104]
2010	SLE	NA	15	Allogeneic BMSC	1 × 10^6^ cells/kg, 1 time	17.2	Overall SLEDAI improvement	mild infections; 0 SAE	NA	[105]
2010	SLE	NA	16	Allogeneic UCSC	1 × 10^6^ cells/kg, 1 time	8.25	Significant reduction in disease activity in all patients, no recurrence	1 AE (nausea); 0 SAE	NCT00698191 (unknown status)	[106]
2012	SLE	NA	35	Allogeneic BMSC/UCSC	1 × 10^6^ cells/kg, 1/2/3 times	21	Overall SLEDAI improvement	5 AE (diarrhea, agranulocytosis, infections); 2 SAE	NCT00698191 (unknown status)	[107]
2014	SLE	NA	40	Allogeneic UCSC	1 × 10^6^ cells/kg, 2 times	12	32.5% major clinical response; 27.5% partial clinical response	4 AE (herpesvirus infection, tuberculosis); 3 SAE	NCT01741857 (unknown status)	[108]
2014	lupus nephritis	NA	81	Allogeneic BMSC/UCSC	1 × 10^6^ cells/kg, 1 time	12	60.5% renal remission; 22.4% renal flare	4 AE (herpes infection, enteritis); 4 SAE	NA	[109]
2017	lupus nephritis	NA	18	Allogeneic UCSC	1 × 10^8^ cells, 1 time	Abandoned	UCSC group: 75% remission; control: 83%	Leucopenia, pneumonia, subcutaneous abscess; 1 SAE	NA	[110]
2025	SLE	I	8	Allogeneic UCSC	1/2/4 × 10^6^ cells/kg, 1 time	12.4	NA	No SAE, 3 infusion‐related AE	NCT03562065 (unknown status)	[111]
2017	RA	Ib/IIa	53	Allogeneic ADSC	1/2/4 × 10^6^ cells/kg, 3 times	6	0–20% ACR50 at month 1	141 AE; 8 SAE, no dose‐related toxicity	NCT01663116 (completed)	[112]
2018	RA	Ia	9	Allogeneic UCSC	2.5/5/10 × 10^7^ cells, 1 time	1	Inflammatory/serological markers improvement; DAS28 score improvement	No dose‐limited AE; no major toxicity	NCT02221258 (completed)	[113]
2019	RA	NA	9	Autologous BMSC	1 × 10^6^ cells/kg, 1 time	12	VAS score decrease	No AE	NCT03333681 (completed)	[114]
2019	RA	I/II	64	Allogeneic UCSC	1 × 10^7^ cells, 1 time	36	Inflammatory/serological markers improvement; DAS28 and HAQ score improvement	No relevant AE	NCT01547091 (unknown status)	[115]
2021	Psoriasis	NA	7	Allogeneic ADSC	0.5 × 10^6^ cells/kg, 3 times	12	28.6% reached and maintained a PASI‐50	16 AE, 1 SAE (no relevant)	NCT03265613 (completed)	[116]
2022	Psoriasis	I/IIa	17	Allogeneic UCSC	1.5/2.0/2.5/3.0 × 10^6^ cells, 4 times	6	47.1% had at least 40% improvement in the PASI score	No obvious AE	NCT03765957 (unknown status)	[117]
2021	Atopic dermatitis	NA	5	Allogeneic BMSC	2.5/5.0 × 10^7^ cells, 6 times	9.5	80% patients reach EASI‐50	0 SAE	NA	[118]
2024	Atopic dermatitis	I/II	20	Allogeneic BMSC	0.5/1 × 10^6^ cells, 3 times	3	Higher proportion of an EASI‐50 response compared with placebo group	No relevant SAE	NCT04179760 (completed)	[119]
2017	Pulmonary fibrosis	I	9	Allogeneic BMSC	20/100/200 × 10^6^ cells, 1 time	15	NA	No relevant SAE	NCT02013700 (terminated)	[120]
2020	Pulmonary fibrosis	I/IIA	10	Allogeneic BMSC	2 × 10^8^ cells, 8 times	10	FVC and DLCO improvements	No relevant AE	NCT02594839 (completed)	[121]
2016	Cirrhosis	II	72	Autologous ADSC	5 × 10^7^, 1/2 times	6	Histologic fibrosis and liver function improvements	Mild fever, no related tumor	NA	[122]
2021	Cirrhosis	NA	219	Allogeneic UCSC	0.5 × 10^6^/kg, 3 times	13–75	Overall survival rate and liver function improvements	Mild fever, no higher risk in long‐term toxicity	NCT01220492 (completed)	[123]
2025	Cirrhosis	I	186	Allogeneic UCSC	0.5/1/1.5/2 × 10^8^ cells, 1 time and ½ × 10^8^ cells, 3 times	1	Improvements in Child‐Pugh scores, model for end‐stage liver disease scores, liver function markers, and quality‐of‐life metrics	No SAE, no dose‐limiting toxicities	NCT05227846 (unknown status) and NCT05984303 (unknown status)	[124]

Abbreviations: ADSC, adipose‐derived stem cells; AE, adverse events; BMSC, bone marrow‐derived stem cells; DAS28, disease activity score for 28 joints; DLCO, diffusing capacity of the lung for carbon monoxide; EASI, eczema area and severity index; FVC, forced ventilation capacity; HAQ, health assessment questionnaire; NA, not available; PASI, psoriasis area and severity index; SAE, severe adverse events; UCSC, umbilical cord stromal cells; WJSC, Wharton's jelly‐derived stromal cells.

Systemic infusion of MSCs has emerged as a clinically validated approach for steroid‐refractory acute GVHD, representing one of the most advanced therapeutic applications in the stem cell field. This modality demonstrates consistent immunomodulatory efficacy, with recent meta‐analyses reporting significantly improved overall and complete response rates, particularly in patients with Grade III–IV acute GVHD [126]. Clinical safety profiles are well‐characterized across large cohorts. The most common adverse events, such as transient nausea and headache, are primarily associated with cryoprotectant use rather than intrinsic cellular toxicity, as supported by existing evidence [83, 89]. Long‐term follow‐up studies have not substantiated treatment‐related mortality or tumorigenic risks [127]. Current protocols [82, 87, 88] predominantly utilize bone marrow‐derived stem cells (BMSCs) administered at standard dose ranges (1–6 × 10^6^ cells/kg), while emerging evidence suggests umbilical cord‐derived sources may exhibit enhanced potency [90, 93, 94]. Notably, in 2024, the United States Food and Drug Administration (US FDA) approved Mesoblast's Ryoncil (Remestemcel‐L) for marketing authorization. This approval marks the first US FDA‐approved MSC therapy and establishes it as the first dedicated treatment for pediatric steroid‐refractory acute GVHD [128].

Regarding systemic MSC infusion in IBD patients, most clinical trials are still in their early phases, and the efficacy remains to be fully established. Current data based on randomized controlled trials (RCTs) (*n* = 82) show that only a few IBD patients achieved temporary clinical or endoscopic remission, despite reduced CDAI scores [104]. While higher dosing regimens were suggested to improve remission rates [100], comparative dose studies found no significant difference [102]. Nevertheless, these studies consistently demonstrate the safety of MSCs in both allogeneic and autologous applications. Adverse events were infrequent and manageable, consisting primarily of transient dysgeusia and allergic reactions associated with dimethyl sulfoxide cryopreservants [97, 100]. Current systemic MSC therapies predominantly utilize bone marrow and umbilical cord sources, with adipose‐derived stem cells (ADSCs) rarely employed. This pattern contrasts with compelling safety data showing that intravenous ADSCs produce no clinical immune reactions [129, 130, 131]. Moreover, trials of locally injected ADSCs have demonstrated reduced CDAI scores and luminal CD remission [132], suggesting that their systemic application warrants exploration beyond fistulizing disease.

Preclinical lupus models demonstrate MSC capacity to reduce autoantibodies, proteinuria, and proinflammatory cytokines while improving renal histology. Early‐phase clinical trials in refractory SLE patients report reduced disease activity [107], decreased proteinuria [109], and serological improvements [107, 108] sustained over 12‐month follow‐up. While long‐term safety data appear favorable with no significant toxicity signals, randomized trials have yielded inconsistent efficacy results [110], necessitating larger controlled studies to establish clinical value. Meanwhile, in RA, systemic MSC infusion has demonstrated promising outcomes in several prospective Phase I/II trials [112, 113, 115]. These studies documented not only serological improvements but also enhanced joint function during follow‐up, with effects persisting for up to 3 years posttreatment [115].

Concurrently, exploratory efforts utilizing systemic MSC therapy for inflammatory skin diseases have yielded promising results. In a Phase 1/2a single‐arm trial, MSCs demonstrated efficacy in psoriasis, with 47.1% of patients achieving ≥40% improvement in psoriasis area and severity index (PASI) scores during the 6‐month follow‐up [117]. This clinical response correlated with significantly restored peripheral blood Treg/Th17 balance in responders, suggesting Treg levels may serve as a predictive biomarker for MSC efficacy [117]. Parallel investigations in atopic dermatitis revealed that even low‐dose MSCs sufficed to improve eczema manifestations compared with placebo controls, with no serious adverse events reported [119].

Fibrotic diseases are notoriously resistant to therapeutic reversal, yet cell‐based therapies offer a promising alternative. Small‐scale studies utilizing MSCs have yielded encouraging results across various etiologies of liver cirrhosis, including alcoholic [122], autoimmune [133], and primary biliary cholangitis [134]. A recent large‐scale prospective RCT involving 219 patients with HBV‐related cirrhosis, followed for up to 75 months, demonstrated that MSC treatment not only significantly improved liver function but also conferred significantly higher overall survival rates compared with the control group [123]. Further mechanistic insight comes from a study employing single‐cell RNA sequencing, which revealed that higher MSC doses elicited stronger immunomodulatory effects and identified MX1‐positive monocytes as a key monocyte subset potentially mediating MSC‐induced immune regulation [124]. By contrast, clinical data on MSC therapy for pulmonary fibrosis remain limited, necessitating further evaluation to determine efficacy and optimal delivery strategies.

#### 3.2.2 Locally Administered Stem Cells

Beyond systemic delivery, targeted administration of stem cells to inflamed or damaged sites offers distinct advantages for diseases with localized pathology. This approach minimizes systemic exposure while maximizing therapeutic concentration at the target tissue, potentially enhancing efficacy and reducing off‐target effects. While extensively studied in FCD, localized stem cell therapy is now being increasingly explored for a variety of other inflammatory conditions. Examples include osteoarthritis, chronic wounds, scleroderma, corneal inflammation, and pancreatitis, particularly in cases where direct application to the affected site is both feasible and physiologically rational [132, 135, 136, 137, 138, 139, 140, 141, 142, 143, 144, 145, 146, 147, 148, 149, 150, 151, 152, 153, 154, 155, 156, 157, 158, 159, 160, 161, 162, 163] (Table 3).

**TABLE 3:** Clinical trials of localized MSC injection.

Year	Indication	Phases	Patients	MSC type	Additional process	Follow up (month)	Efficacy	Adverse event	NCT No. (status)	References
2003	FCD	NA	1	Autologous ADSC	Advancement vaginal flap	3	100% fistula healing rate	NA	NA	[135]
2005	FCD	I	5	Autologous ADSC	/	22(12–30)	75% fistula healing rate	No related AE/SAE	NA	[136]
2009	FCD	II	14	Autologous ADSC	/	12	71% fistula healing rate	11 AE; 2 SAE (unrelated)	NA	[137]
2011	FCD	NA	12	Autologous BMSC	/	12	70% fistula healing rate	No related AE/SAE	NA	[132]
2013	FCD	I/IIa	24	Allogeneic ADSC	/	6	56.3% fistula healing rate	5 related AE; 2 related SAE	NCT01372969 (completed)	[138]
2013	FCD	I	10	Autologous ADSC	/	8	37.5% fistula healing rate	13 AE (unrelated)	NA	[139]
2015	FCD	II	41	Autologous ADSC	/	24	80.8% fistula healing rate	53 AE (unrelated)	NCT01011244 (completed) and NCT01314079 (completed)	[140]
2015	FCD	II	21	Allogeneic BMSC	/	6	66.7% (low‐dose group); 85.7% (medium‐dose group); 28.6% (high‐dose group); 33.3% (placebo group) fistula healing rate	No related AE/SAE	NCT01144962 (completed)	[141]
2016	FCD	III	212	Allogeneic ADSC	/	6.5	50% fistula healing rate	18 related AE; 5 related SAE	NCT01541579 (completed)	[142]
2016	FCD	I/IIa	10	Allogeneic ADSC	/	25	60% fistula healing rate	No SAE	NCT00999115 (completed)	[143]
2018	FCD	NA	9	Autologous ADSC	/	31(21–37)	91% fistula healing rate	NA	NA	[144]
2020	FCD	I	5	Autologous ADSC	Bioabsorbable scaffold (MSC‐MATRIX)	6	0% fistula healing rate	No related AE/SAE	NA	[145]
2021	FCD	NA	4	Allogeneic ADSC	/	6	25% fistula healing rate	3 SAE	NA	[146]
2021	FCD	NA	12	Allogeneic ADSC	/	14.3 (3–10)	66.7% fistula healing rate	No SAE	NA	[147]
2021	FCD	IV	5	Autologous ADSC	/	6	80% (8 w); 20% (6 m) fistula healing rate	No related AE/SAE	NA	[148]
2022	FCD	Ib/IIa	4	Allogeneic BMSC	/	3	NA	No related AE/SAE	NCT04543994 (unknown status)	[149]
2023	FCD	Ib/IIa	18	Allogeneic BMSC	/	12	83% fistula healing rate	No related AE/SAE	NA	[150]
2023	FCD	I	7	Allogeneic BMSC	/	6	83% fistula healing rate	No related AE/SAE	NA	[151]
2024	FCD	I/II	10	Allogeneic BMSC	/	26	20% (6 m); 20% (13 m); 20% (26 m) fistula healing rate	3 AE	NA	[152]
2024	FCD	NA	17	Allogeneic UCSC	/	24	NA	No related AE/SAE	NA	[153]
2016	KOA	I/II	30	Autologous BMSC	Hyaluronic acid	12	WOMAC, VAS and MRI dose‐dependent improvement	No AE/SAE	NCT02123368 (completed)	[154]
2019	KOA	I/II	40	Allogeneic UCSC	/	12	WOMAC, VAS dose‐dependent improvement	Acute synovitis, symptomatic knee effusion, No SAE	NCT02580695 (completed)	[155]
2020	KOA	II	60	Autologous BMSC	Platelet‐rich plasma	12	No statistical significances	No AE/SAE	NCT02365142 (unknown status)	[156]
2023	KOA	III	146	Allogeneic BMSC	/	12	WOMAC and MRI improvement	5 related AE (swelling, pain)	NA	[157]
2023	KOA	III	261	Autologous ADSC		6	WOMAC, VAS improvement	No related SAE	NCT03990805 (completed)	[158]
2025	KOA	NA	30	Allogeneic UCSC	/	12	WOMAC, SF‐36 improvement	No related AE/SAE	NA	[159]
2017	Atopic dermatitis	I/IIa	34	Allogeneic UCSC	/	3	55% reach EASI‐50 in high dose group	Local reactions, infection, gastrointestinal disorder; 0 SAE	NCT01927705 (completed)	[160]
2022	Diabetic ulcer	I	14	Allogeneic UCSC	Intravenous MSC injection	36	>95% ulcer healing within 1.5 months	2 related AE (fever), all survived without amputation at year 3	NA	[161]
2023	Psoriasis	I	5	Allogeneic ADSC	/	6	Skin thickness, erythema, and scaling of the plaques, PASI score were decreased	No major SE	NA	[162]
2018	RA	I/II	30	Autologous BMSC	/	12	WOMAC, VAS, time to jelling and pain‐free walking distance improvement	No related AE/SAE	NCT01873625 (completed)	[163]

Abbreviations: FCD, fistulizing Crohn's disease; KOA, knee osteoarthritis; VAS, visual analogue scale; WOMAC, Western Ontario and McMaster Universities Osteoarthritis Index.

Fistula, occurring in 20–30% of CD patients [164, 165] and increasing with disease duration [166], represents a challenging complication. Despite advances in biological agents and surgical interventions, fistulizing CD remains difficult to manage and requires constant medical care. Traditional surgical approaches, such as drainage, fistulotomy, and ligation, have limited efficacy for inflammatory fistulas [167].

Limited trials using BMSCs for FCD have shown high rates of fistula healing with no related adverse effects reported [132, 141]. Long‐term follow‐up data demonstrate durable responses, with autologous BMSC recipients maintaining 88% fistula relapse‐free survival at 1 year and 37% over 4 years [168], while allogeneic BMSC trials achieved 100% closure in medium‐dose cohorts at 4‐year follow‐up [169]. Compared with BMSCs, ADSCs are favored due to their abundance in adipose tissue [170] and less invasive harvesting methods, for example, liposuction [171]. Key studies include (1) Garcia‐Olmo et al.’s 2003 case report [135] and subsequent Phase I trial [136]. (2) Cho et al.’s studies [139, 140, 172] demonstrating dose‐dependent efficacy and long‐term safety. (3) Recent Phase IV study expanding ADSCs use to children with refractory FCD [148]. Commercialized ADSC products include Darvadstrocel [142, 173, 174] (the first advanced MSC therapy approved in the European Union, eliminating the need for donor harvesting procedures) and Remestemcel‐L [150, 151, 152] (demonstrating safety and efficacy in Phase I/II studies). Recent studies have also explored local injections for luminal CD and UC with promising initial results [149, 153]. The accumulating evidence from clinical trials and commercialized products demonstrates that localized MSC therapy, particularly using ADSCs, represents a safe and promising treatment option for FCD. Notably, some fundamental questions regarding fistula pathophysiology and interactions between MSCs and their microenvironment remain unresolved. More fundamentally, the pathophysiological environment of fistulae remains incompletely understood. Current hypotheses suggest that CD‐associated fistulae originate from epithelial defects driven by multifactorial processes, including destructive inflammation, epithelial‐to‐mesenchymal transition, matrix metalloproteinase activity, and soluble mediator dysregulation [175, 176]. Critically, the role of MSCs in fistula pathogenesis and microenvironment modulation remains understudied, partly due to the inadequacy of current animal models. Although porcine models are anatomically relevant to human anal structures, they face practical constraints such as procedural complexity, high costs, and extended timelines [177]. These factors limit their widespread implementation.

In knee osteoarthritis (KOA) research, intra‐articular MSC injections remain a major focus, spurring numerous high‐quality RCTs. A pivotal RCT involving 146 patients demonstrated that allogeneic BMSCs conferred protective effects. These included improvements in standardized outcome measures (WOMAC, VAS scores) and mitigating further cartilage degradation on imaging during extended follow‐up [157]. MSCs are sometimes combined with established therapies such as platelet‐rich plasma (PRP). Although studies have not yet demonstrated statistically significant additive benefits over MSC monotherapy, these combinations may enhance responses in some patients [156]. Similarly, compared with hyaluronic acid alone, adjunctive MSC therapy, particularly at higher doses, yields significantly greater improvements in WOMAC scores and modest radiological improvements [154]. Notably, these clinical benefits persisted throughout 4‐year follow‐up while maintaining excellent safety profiles [178]. Furthermore, repeated MSC administrations have shown superior efficacy to single‐dose regimens [155]. Recent studies of mature commercial cell preparations have also yielded promising results, with MSC interventions outperforming placebo and even corticosteroid controls [157, 159].

Beyond FCD and KOA, localized MSC therapy has demonstrated potential across other inflammatory conditions, especially skin lesions. A preliminary clinical study (*n* = 34) investigating subcutaneous MSC injections for atopic dermatitis reported favorable safety profiles alongside promising therapeutic trends, with great improvements in clinical symptoms [160]. Similarly, in psoriasis patients receiving intralesional ADSC injections, treatment proved safe with no major adverse events, while yielding clinical improvements including reduced plaque thickness, erythema, scaling, and PASI scores over 6‐month follow‐up [162]. Parallel findings emerged in diabetic foot ulcer patients with peripheral arterial disease, where combined topical/intravenous UCSC administration achieved >95% ulcer healing within 1.5 months and maintained 100% amputation‐free survival at 3 years, with only transient fever reported as a treatment‐related adverse event [161]. These findings collectively reinforce the emerging view that targeted MSC delivery to localized inflammatory areas (including cutaneous, vascular, and enteric sites) can achieve therapeutic benefits while maintaining excellent safety parameters. Though more clinical evidence is required, the anatomical and immunological specificity of such approaches may explain their superior risk–benefit ratios compared with systemic administration for focal disease manifestations.

In conclusion, these clinical explorations demonstrate that localized MSC administration is optimally suited for diseases with defined inflammatory foci, where direct cell delivery to affected sites enables more rapid and potent therapeutic effects compared with systemic infusion. This approach further favors ADSCs over BMSCs, reflecting their practical advantages in accessibility and niche‐specific reparative functions for targeted interventions. The successful development of approved products, such as Remestemcel and Darvadstrocel, marks a significant milestone in translating stem cell therapy from the bench to the bedside. The emerging exploration of local MSC treatment in fistula and luminal disease further expands the potential therapeutic scope; however, additional research is needed to establish its efficacy in these applications fully.

### 3.3 Sources of Stem Cells

Stem cell sourcing strategies fundamentally dictate therapeutic efficacy and clinical applicability in disease management. Classification encompasses three interdependent taxonomic dimensions: species origin (xenogeneic vs. allogeneic vs. syngeneic), donor compatibility (autologous vs. allogeneic), and tissue derivation (e.g., mesenchymal vs. hematopoietic niches). For HSCs, which are primarily harvested from bone marrow or mobilized peripheral blood, the critical factor is ensuring immunological compatibility between donor and recipient. Autologous HSCs eliminate graft rejection risks but retain genetic susceptibility footprints, whereas allogeneic HSCs offer curative potential for monogenic defects at the cost of GVHD risk. These divergent immunological profiles directly impact engraftment kinetics: autologous grafts typically achieve faster reconstitution, while allogeneic variants require prolonged immunosuppression but enable complete immune reset. In contrast, MSCs present a different classification paradigm. While autologous versus allogeneic distinctions remain relevant, the inherently low immunogenicity of MSCs shifts clinical focus toward their diverse tissue origins, including bone marrow, adipose tissue, umbilical cord, and other mesenchymal sources, each potentially offering distinct therapeutic characteristics. Simultaneously, contemporary advances extend beyond natural origins to engineered pluripotent sources. As a rapidly evolving category, iPSCs demonstrate exceptional reprogramming plasticity and genetic editability. These synthetic stem cells are poised to emerge as a unique, standardized therapeutic reservoir for many inflammatory diseases in the near‐future clinical translation landscape.

#### 3.3.1 Autologous HSCT

Autologous HSCT is generally preferred over allogeneic transplantation in treatment of some inflammatory diseases, primarily due to its lower risk profile and the absence of GVHD [179]. Unlike allogeneic HSCT's renewal of hematopoietic systems, autologous HSCT functions as an immunological reset: it eradicates pathogenic immune memory through intensive conditioning regimens, followed by infusion of purified HSCs to reconstitute a tolerant immune repertoire, thereby fundamentally reprogramming immune tolerance pathways.

For IBD patients, supportive results have been observed in some clinical trials, showing a generally acceptable outcome [179, 180, 181, 182, 183, 184, 185, 186, 187, 188, 189] (Table 4). Key clinical trials: (1) “ASTIC trial” is the first RCT evaluating autologous HSCT in refractory CD [190]. Only eight (34.8%) patients in the HSCT group were free of active disease on endoscopy and radiology at the final assessment, while 76 serious adverse events and one death occurred [185]. It has been pointed out that strict targets and high‐dose conditioning regimens may have influenced outcomes [191]. Apart from that, secondary endpoints showed statistically significant improvements in clinical remission and endoscopic disease activity [185]. Similarly, long‐term observations have demonstrated regained drug sensitivity and an acceptable long‐term safety profile [179, 187]. (2) Infections remain the primary complication and adverse effect throughout the HSCT procedure, attributed mainly to the high‐dose cyclophosphamide used in conditioning regimens [192]. Low‐dose cyclophosphamide studies have demonstrated lower hematological toxicity and fewer complications [183, 188]. Additionally, remission rates were similar to or better than those achieved with high‐dose regimens [188], although long‐term data remain unavailable. (3) A parallel‐group, controlled trial using low‐dose cyclophosphamide/G‐CSF mobilization and reduced intensity conditioning [193] halted due to unexpected serious adverse events. These adverse events contained nine suspected unexpected SAEs in six (46%) patients, including renal failure due to thrombotic microangiopathy (three cases) and one death due to pulmonary veno‐occlusive disease [189].

**TABLE 4:** Clinical trials of autologous HSCT for patients with refractory inflammatory disease.

Year	Indication	Phases	Patients	Mobilization	CD34 selection	Immune conditioning	Follow up (month)	Efficacy	Adverse event	NCT No. (status)	References
2003	Refractory CD	NA	2	CTX (2.0 g/m^2^) and G‐CSF (5 mg/kg/d)	+	CTX (200 mg/kg) and equine ATG (90 mg/kg)	15	2/2 clinical remission for 12 months	No unexpected AE/SAE	NA	[180]
2005	Refractory CD	NA	12	CTX (2.0 g/m^2^) and G‐CSF (10 µg/kg/d)	+	CTX (200 mg/kg) and equine ATG (90 mg/kg)	7–37	11/12 clinical remission at the average of 18.5 months; 1 relapse after 15 months	1 Mallory–Weiss hematemesis	NA	[181]
2008	Refractory CD	NA	4	CTX (1.5 g/m^2^) and G‐CSF (10 mg/kg/d)	−	CTX (200 mg/kg) and rabbit ATG (7.5 mg/kg)	11–20	4/4 clinical remission at 3 months, 3/4 at 12 months; 2/3 endoscopic remission at 3 months, 3/4 at 12 months	1 perianal abscess; 1 pleural and pericardial effusions; 1 BK virus‐related macrohematuria	NA	[182]
2010	Refractory CD	NA	24	CTX (2.0 g/m^2^) and G‐CSF (10 µg/kg/d)	+	CTX (200 mg/kg) and equine ATG (90 mg/kg) or rabbit ATG (6 mg/kg)	60	Rapid and significant CDAI improvement; clinical relapse‐free survival rate after HSCT is 91% at 1 year, 63% at 2 years, 57% at 3 years, 39% at 4 years, and 19% at 5 years	Fever and bacteremia during hospitalization; 10 infections during the first year after HSCT	NCT00278538 (completed)	[183]
2012	Refractory CD	NA	12	CTX (2.0 g/m^2^) and G‐CSF (5 µg/kg/d)	+	CTX (200 mg/kg)	6–123	4/8 clinical remission at 3 months; 5/9 endoscopic remission at 9.1 months (average); 7/9 relapse	4 SAE during mobilization (prolonged neutropenic fever, reversible acute renal failure, vaginal bleeding, urinary retention)	NA	[184]
2015	Refractory CD	III	45	CTX (2.0 g/m^2^) and G‐CSF (10 µg/kg/d)	−	CTX (200 mg/kg) and rabbit ATG (7.5 mg/kg)	7–37	8/23 free of active disease on imaging at 1 year; 8/23 clinical remission at 3 months	76 SAE in HSCT group; 38 SAE in control group; 1 death	NCT00297193 (terminated)	[185]
2016	Refractory CD	NA	26	CTX (2.0 g/m^2^) and G‐CSF (10 µg/kg/d)	−	CTX (200 mg/kg) and rabbit ATG (7.5 mg/kg)	12	N/A	16/26 febrile neutropenia during mobilization; 20/21 febrile neutropenia, 3/21 worsening of perianal disease, 6/21 ATG reaction, 12/21 mucositis during conditioning and transplantation; 16/21 febrile neutropenia and 1 death for CMV during long‐term observation	NA	[186]
2017	Refractory CD	NA	29	CTX (2.0 g/m^2^) and G‐CSF (10 µg/kg/d)	−	CTX (200 mg/kg) and rabbit ATG (7.5 mg/kg)	60	70% of patients achieved drug‐free clinical remission at 6 months, and 61% at 1 year, 52% at 2 years, 47% at 3 years, 39% at 4 years, and 15% at 5 years	23/29 febrile neutropenia during conditioning and transplantation; 14/29 noninfectious complications during conditioning and transplantation; 11/29 herpes virus infection; 1 death from CMV infection, 8 symptomatic adrenal insufficiency during long‐term observation	NA	[187]
2017	Refractory CD	NA	14	CTX (60 mg/kg/d) and G‐CSF (10 µg/kg/d)	−	CTX (200 mg/kg) and rabbit ATG (6.5 mg/kg)	1	13/14 clinical remission at 1 month	Diarrhea and infections during conditioning	NCT03000296 (unknown status)	[188]
2018	Refractory CD	NA	82	N/A	NA	NA	6–174	Clinical remission 68% at a median follow‐up of 41 months; 54% treatment‐free survival at 1 year	22/82 infection requiring treatment, 9/82 secondary autoimmune disease, 5/82 new malignancy one year after HSCT; 1 death for CMV infection	NA	[179]
2024	Refractory CD	NA	20	CTX (1.0 g/m^2^) and G‐CSF (5 µg/kg/d)	−	Fludarabine (125 mg/m^2^) and CTX (120 mg/kg) and rabbit ATG (7.5 mg/kg)	12	Halted; 43% remission rate in the intervention group, none in the control group	9 SAEs in six patients in the intervention group, including renal failure due to thrombotic microangiopathy in three participants, and one death due to pulmonary veno‐occlusive disease	NA	[189]
1997	MS / SLE / RA	NA	10	CTX (1.0 g/m^2^) and G‐CSF (5 µg/kg/d)	+	CTX (200 mg/kg), methylprednisolone (4 g) and ATG (90 mg/kg) or body irradiation (1200 cGy), methylprednisolone (4 g), CTX (120 mg/kg)	5–17	All patients have demonstrated stabilization or improvement	NA	NA	[194]
2000	SLE	NA	7	CTX (2.0 g/m^2^) and G‐CSF (10 µg/kg/d)	+	CTX (200 mg/kg), methylprednisolone (1 g), and equine ATG (90 mg/kg)	12–40	All patients were free from signs of active lupus	7 fever, 7 fluid retention, 2 dermatomal herpes zoster, 1 pneumonia	NA	[195]
2006	SLE	II	50	CTX (2.0 g/m^2^) and G‐CSF (5 µg/kg/d)	+	CTX (200 mg/kg), and equine ATG (90 mg/kg)	29–90	84% overall 5‐year survival rate, 50% disease‐free survival at 5 years	1 death, 1 pneumonia, 14 bacteremia transplantation, etc	NCT00271934 (completed)	[196]
2009	SLE	I/II	7	CTX (0.5–1.0 g/m^2^) and G‐CSF (10 µg/kg/d)	+	CTX (200 mg/kg), methylprednisolone (1 g), and rabbit ATG (30 mg/kg)	1–96	6/7 achieved long‐lasting clinical and serologic remissions	NA	NCT00742300 (unknown status)	[40]
2017	SLE	NA	24	NA	NA	NA	120	21/24 achieved remission, 2/24 partial remissions at 6 months; 86.0% 10‐year overall survival rate, 86.0% 10‐year remission survival rate	NA	NA	[197]
2019	Lupus nephritis	NA	22	CTX (2.0 g/m^2^) and G‐CSF (10 µg/kg/d)	+	CTX (150 mg/kg), and rabbit ATG (7.5 mg/kg)	60–80	82% completed remission, 5% partial remission	Several grade 1/2 complications; no grade 4/5 complications occurred	NCT03828071 (completed)	[198]
2011	SSc	II	19	CTX (2 g/m^2^) and filgrastim (10 µg/kg/d)	−	CTX (200 mg/kg), and rabbit ATG (6.5 mg/kg)	12	0 disease progression in HSCT group; 88.9% in control group	2 arrhythmias, 2 volume overload, 1 CMV reactivation	NCT00278525 (completed)	[199]
2014	SSc	NA	156	CTX (4 g/m^2^) and filgrastim (10 µg/kg/d)	+	CTX (200 mg/kg), methylprednisolone (1 mg/kg), and rabbit ATG (7.5 mg/kg)	48	HSCT group has better long‐term event‐free survival	19 deaths and 3 irreversible organ failures in HSCT group; 23 deaths and 8 irreversible organ failures in control group	NA	[200]
2018	SSc	II/III	75	G‐CSF	+	Fractionated total‐body irradiation (800 cGy) + CTX (120 mg/kg), and equine ATG (90 mg/kg)	54	79% event‐free survival rate at 54 months in HSCT group; 50% in the CTX group	Increased SAE/AE rate in HSCT group	NCT00114530 (completed)	[201]
2019	SSc	NA	19	CTX (4 g/m^2^) and filgrastim (10 µg/kg/d)	−/+	CTX (200 mg/kg)	69.6	5‐year PFS rates of the CD34+/CD34− group were 81.8 and 50%	11 viral infections; 8 grade 3–4 AE	NA	[202]

Abbreviations: ATG, antithymocyte globulin; CMV, cytomegalovirus; CTX, cyclophosphamide; G‐CSF, granulocyte colony‐stimulating factor; SSc, systemic sclerosis.

Similar results have been achieved in the review of clinical trials for other inflammatory diseases, such as SLE. Autologous HSCT has demonstrated particular promise for refractory cases, with clinical studies reporting sustained disease remission and serological improvement in a substantial proportion of patients [195, 196]. The therapeutic approach also appears to induce immune system resetting, as evidenced by the disappearance of pathogenic autoantibodies and the restoration of immunological tolerance [40]. While infection risks remain a concern during the peri‐transplantation period [195, 196], long‐term follow‐up data suggest durable responses in some patients, with a subset achieving prolonged drug‐free remission [40]. However, the relatively high relapse rates highlight the need for optimized conditioning protocols and posttransplant maintenance strategies [197].

In systemic sclerosis (SSc), autologous HSCT has also been investigated as a potential treatment strategy. The “ASSIST trial” reported improvements in skin involvement and forced vital capacity at 12‐month follow‐up among transplanted patients [199]. A study comparing CD34^+^‐selected versus unselected graft strategies showed that the CD34^+^‐selected group was associated with better 5‐year progression‐free survival (81.8 vs. 50%), greater improvement in skin thickening and pulmonary function, and a comparable profile of adverse events [202]. A large phase III RCT (*n* = 156) confirmed that although HSCT increased treatment‐related mortality in the first year, it provided significant long‐term event‐free survival benefits [200]. Additionally, myeloablative conditioning regimens have been explored in SSc patients, demonstrating long‐term advantages including improved event‐free survival [201]. These regimens were reported to have lower treatment‐related mortality and reduced posttransplant dependence on DMARDs compared with nonmyeloablative approaches [201]. However, the suitability of highly intensive myeloablative protocols involving total body irradiation for all SSc patients requires further evaluation.

While autologous HSCT shows promise in treating refractory inflammatory diseases, particularly in achieving clinical remission, its widespread adoption faces significant challenges due to safety concerns. The mixed results from clinical trials, including the “ASTIC trial,” highlight the critical need to optimize conditioning regimens. Although low‐dose cyclophosphamide protocols show the potential to reduce complications while maintaining efficacy, recent trials reporting unexpected serious adverse events underscore the importance of careful patient selection and rigorous monitoring. Future research should focus on establishing optimal conditioning protocols that balance therapeutic efficacy and safety.

#### 3.3.2 Allogeneic HSCT

Although there are limited clinical trials that rigorously discuss the efficacy of allogeneic HSCT in the treatment of refractory inflammatory diseases, the occasional case of patients with inflammatory diseases undergoing HSCT for coexisting hematologic tumors has demonstrated great therapeutic potential [21, 203, 204, 205, 206]. Some researchers suggest that allogeneic transplantation may surpass autologous transplantation in terms of treatment completeness [207]. This allogeneic approach achieves durable remission in a substantial proportion of patients, with many maintaining long‐term drug‐free disease control [203, 205], reflecting a level of therapeutic completeness rarely attainable through autologous transplantation. The fundamental efficacy derives from the replacement of genetically susceptible immune cells, which potentially corrects underlying immunodeficiencies. However, this promise is counterbalanced by substantial clinical risks, including frequent graft‐versus‐host reactions, both acute and chronic forms, alongside significant treatment‐related mortality, predominantly from opportunistic infections.

Efforts are continuously being made to optimize this procedure. Current evidence indicates that both myeloablative and reduced‐intensity conditioning regimens are viable options, although optimal selection criteria remain debated [208, 209]. Notably, a pilot study using reduced‐intensity conditioning for refractory CD reported sustained remission in most patients during a 5‐year follow‐up [210], suggesting that therapeutic effects may derive not only from HSC engraftment but also from conditioning‐induced immunomodulation. Thus, given this complex risk–benefit landscape, current application remains appropriately confined to specific clinical scenarios, particularly patients with concurrent hematological malignancies.

Notably, another condition where allogeneic HSCT plays a vital and potentially curative role is very early‐onset IBD (VEO‐IBD). Unlike typical UC and CD, VEO‐IBD is often associated with underlying PID, causing patients to develop severe symptoms in infancy and early childhood. Many of these patients are refractory to conventional treatments and face a high risk of morbidity and mortality [27]. In such cases, allogeneic transplantation is highly recommended and may serve as the only potential cure [25, 26, 28, 29, 208, 209, 211, 212, 213, 214, 215, 216, 217, 218, 219, 220, 221, 222, 223] (Table 5). The potential for allogeneic transplantation to induce more complete responses warrants further investigation through well‐designed clinical trials, which could open new avenues for treating severe conventional‐therapy‐refractory IBD.

**TABLE 5:** Major published case series on allogeneic HSCT for primary immunodeficiencies.

Year	Monogenic mutation/PIDD	Patients	Age for HSCT	Efficacy	Adverse event	References
2009	IL‐10R deficiency	1	NA	Clinical remission during follow‐up (>1 year); anal fistulas resolved	Acute GVHD	[25]
2012	IL‐10R deficiency	5	8 y 3 m (10 m–13 y 9 m)	4/5 clinical remission up to 2 years, 1/5 improved	1 acute GVHD, 1 chronic GVHD	[211]
2013	IL‐10R deficiency	3	3 y 2 m; 3 y 11 m; 1 y 2 m	Clinical remission; fistula resolved	NA	[212]
2014	IL‐10R deficiency	2	5 y; 10 y	1 clinical remission >7 months, 1 clinical remission >16 months	No acute/chronic GVHD	[213]
2015	IL‐10R deficiency	1	7 m	Clinical remission; fistula resolved	No acute/chronic GVHD	[214]
2020	IL‐10R deficiency	13	NA	NA	6 GVHD; 5 deaths	[209]
2011	CGD	1	6 y	Clinical remission during follow‐up (>40 days)	NA	[215]
2016	CGD	1	20 y	Clinical and endoscopic remission during follow‐up (>16 months)	No acute/chronic GVHD	[216]
2019	CGD	49	11 y (1–26 y)	Clinical remission in all 33 survived patients	38% acute GVHD, 18% chronic GVHD	[28]
2011	XIAP deficiency	1	5y8 m	Clinical remission	Acute GVHD	[208]
2013	XIAP deficiency	3	NA	NA	NA	[26]
2015	XIAP deficiency	1	7 y	Endoscopic remission >55 days; clinical remission >11 months	Acute and chronic GVHD	[217]
2021	XIAP deficiency; IL‐10R deficiency	4	4 y; 8 y; 20 y; 7 m	Clinical and endoscopic remission	NA	[29]
2016	LRBA deficiency	1	14 y	Clinical remission	No acute/chronic GVHD	[218]
2017	LRBA deficiency	1	12 y	Clinical remission	No acute/chronic GVHD	[219]
2008	CD3γ‐deficiency	1	10 m	Clinical improved; fistula improved	Died of infection day 50 after second transplant	[220]
2019	G6PC3 deficiency	1	20 y	Clinical remission during follow‐up (>2.5 years)	Grade II skin GVHD	[221]
2021	G6PC3 deficiency	3	11 y, 14 y, 17 y	Clinical remission	1 autoimmune hemolytic anemia	[222]
2021	NCF4 deficiency	1	9 y	Clinical and endoscopic remission during follow‐up (>6 months)	NA	[223]

Abbreviations: CGD: chronic granulomatous disease; G6PC3: glucose‐6‐phosphatase 3; LRBA: lipopolysaccharide‐responsive beige‐like anchor; NA, not available; NCF4: neutrophil cytosolic factor 4; PIDD: primary immunodeficiency diseases; XIAP: X‐linked inhibitor of apoptosis.

Patient selection for HSCT in VEO‐IBD requires careful consideration of underlying genetic etiology, as not all patients are suitable candidates, and not all monogenic defects are amenable to correction through this intervention. To date, approximately 80 monogenic causes of VEO‐IBD have been documented [224], yet only a subset of these genetic defects can be effectively addressed through allogeneic HSCT. Monogenic disorders involving components of innate and adaptive immunity represent the most promising candidates for allogeneic HSCT, as transplanted hematopoietic cells can reconstitute defective immune functions. Conversely, patients with epithelial barrier‐related mutations are theoretically unlikely to benefit from allogeneic HSCT [225], since transplanted hematopoietic cells cannot restore epithelial barrier integrity or function.

C1q deficiency is another notable example. This rare congenital immunodeficiency, characterized by defective clearance of immune complexes, exemplifies a monogenic disorder amenable to allogeneic HSCT [226]. A recent multicenter retrospective study of allogeneic HSCT for C1q deficiency (18 patients/20 transplant procedures) demonstrated that HSCT led to the regression of autoimmune features and enabled the discontinuation of immunosuppressive therapy in 11 patients (follow‐up: 3–84 months), while five patients died of transplantation‐related complications [31]. Significantly worse overall survival can be observed in patients with severe neurological/renal involvement [31]. This underscores HSCT's curative potential for other immune‐reconstitution inflammatory disorders while highlighting the critical need for careful patient selection.

#### 3.3.3 MSCs Derived From Different Tissues

MSCs are present in various tissues, such as bone marrow [227], adipose tissue [228], and umbilical cord [229]. While MSCs from different sources share broadly similar characteristics and functions, subtle differences exist. For instance, BMSCs express higher levels of immunosuppressive cytokines [230], ADSCs demonstrate stronger proliferative capacity [231], and placenta‐derived stem cells exhibit the most significant potential for hepatogenic differentiation [232]. Despite these nuanced variations, the fundamental properties of MSCs remain largely consistent across different tissue origins. Meanwhile, in clinical translation, practical feasibility often takes precedence over functional comparisons. Bone marrow sourcing requires invasive aspiration procedures, whereas ADSCs offer logistical advantages through minimally invasive liposuction with high cell yield. Placental and umbilical sources provide relatively standardized products through cord blood banks, despite ethical considerations. Current therapeutic preferences are often rooted in historical conventions: BMSCs for systemic immunomodulation via intravenous infusion, ADSCs for localized therapy, and placental derivatives for scalable manufacturing. While acknowledging their empirical value, these preferences must be applied cautiously to ensure that evidence‐based optimization is prioritized over anecdotal experience. Further systematic comparisons of MSCs from various tissue sources, particularly focusing on their immunomodulatory properties, expansion potential, and therapeutic efficacy in disease‐specific contexts, would help optimize source selection for specific clinical applications.

#### 3.3.4 TSCs and Organoids

TSCs and their derived organoid technologies are gradually becoming a key platform for inflammatory disease research. Their value is not only limited to intestinal diseases but also extends to the mechanism analysis and regenerative treatment of inflammatory lesions in multiple systems such as the skin and joints.

Intestinal epithelial cells (IECs) and ISCs play critical roles in supporting mucosal regeneration and maintaining intestinal homeostasis. ISCs serve as the primary engines of epithelial renewal, continuously generating progenitor cells that differentiate into the diverse IEC lineages essential for mucosal integrity. Mainly through Wnt and Notch signaling‐mediated coordination, ISCs dynamically maintain crypt‐villus architecture while responding to injury through accelerated proliferation and differentiation [233, 234]. Intervention or treatment of IBD targeting or utilizing ISCs appears to be a foreseeable path; however, the lack of suitable culture systems and limited understanding of the ISC niche hinder early research on the intestinal epithelium. However, the field advanced significantly when Sato et al. successfully simulated the ISC niche and constructed crypt‐villus structures in vitro [235], marking the beginning of organoid research.

3D culture techniques enable the continuous and robust expansion of ISCs in vitro without the need for mesenchymal cell support [235]. Organoids have become a reliable platform for studying IBD pathophysiology [236, 237, 238] and testing new therapeutics [239, 240]. Transplantation of cultured colon organoids into damaged mouse colon demonstrated long‐term regeneration of normal epithelial cells (over 6 months) and a significant improvement in symptoms of DSS‐induced colitis [241]. Similar successful transplantations have been achieved in the small intestine [242]. Paneth cells in the small intestine and deep crypt secretory cells in the colon play crucial roles in building regulatory niches and ensuring long‐term survival of ISC grafts [243, 244].

Meran et al. reported a groundbreaking attempt to create hybrid small‐intestine grafts [245]. Their approach involved: (1) Integrating patient‐derived intestinal organoids propagated from biopsy samples into decellularized intestinal scaffolds. (2) Using intestinal scaffolds surgically resected from patients with intestinal failure. (3) Transplanting these hybrid grafts into mice. Biophysical assessments confirmed the flexibility and durability of the decellularized scaffolds, which retained extracellular matrix constituents and proteins similar to those found in native human intestine. This work represents a significant step toward the clinical translation of organoid technology and regenerative medicine, laying the groundwork for future allogeneic transplantation studies.

Beyond ISCs, therapeutic applications of TSCs from diverse sources are gaining momentum. Epidermal stem cells (EpSCs) exhibit significant promise for inflammatory skin disorders Their successful use in restoring skin integrity in epidermolysis bullosa [246] demonstrates critical barrier repair capabilities applicable to conditions like atopic dermatitis and psoriasis. Furthermore, EpSCs harness immunoregulatory properties to resolve dysregulated inflammation in wound models [246], positioning them as key modulators of pathological skin microenvironments. Similarly, recently developed cartilage organoids have achieved successful implantation in primate articular cartilage defect models [247], showing potential to alleviate joint degeneration in RA. Notably, iPSC‐derived liver organoids have also demonstrated therapeutic efficacy in hepatic fibrosis models [248]. Upon transplantation, these organoids not only regenerate functional liver tissue but also polarize CD163^+^ M2 macrophages [248]. This polarization represents a key antifibrotic mechanism, leading to significant fibrosis regression and functional recovery. These advances in TSCs and organoids fundamentally rely on innovations in iPSCs technology, which enables the standardized production and genetic customization of therapeutic cells.

#### 3.3.5 iPSCs

iPSCs represent a fundamentally different approach to stem cell therapeutics, functioning as engineered pluripotent cells rather than naturally occurring tissue‐resident stem cells. iPSCs are generated by reprogramming terminally differentiated somatic cells back to a pluripotent state through the induced expression of defined transcription factors [249]. This reprogramming capability, achievable through multiple methodological approaches, positions iPSCs as a potentially unlimited and standardizable source of therapeutic cells, with demonstrated capacity to differentiate into any tissue type under appropriate culture conditions. The versatility of iPSCs technology is exemplified by established protocols for generating high‐purity hematopoietic lineages, including definitive erythrocytes, megakaryocytes, and bone marrow progenitor cells [250]. These protocols have promising applications in scalable blood product manufacturing for transfusion medicine.

Importantly, preclinical studies demonstrate that iPSCs exhibit therapeutic efficacy comparable to naturally derived stem cells [33, 54] and activate analogous therapeutic pathways through mechanisms such as TSG‐6 signaling [33, 251, 252]. These findings support the therapeutic potential of iPSC‐based interventions in inflammatory disease treatment.

Beyond therapeutic potential, iPSC technology provides an unparalleled platform for investigating individualized disease mechanisms and associated genetic susceptibilities. This is particularly impactful for rare conditions like VEO‐IBD, where patient biopsies are scarce. By reprogramming somatic cells from affected individuals, researchers establish patient‐specific IL‐10RB‐deficient models that recapitulate pathological processes inaccessible through conventional approaches [253, 254]. Furthermore, through iPSC‐derived ISC, an in vitro model of physiologically responsive human small intestinal and colonic organoids has been successfully developed [255]. This modeling system has been confirmed to be suitable for studying barrier dysfunction and the role of epithelium in IBD, representing a platform to assess the effects of various stimuli and environmental factors [255, 256].

The landmark CYP‐001 trial represents the first completed clinical study of iPSC‐derived cells in inflammatory diseases. This phase I trial investigated intravenous infusion of iPSC‐derived MSCs (CYP‐001) in 15 patients with steroid‐resistant acute GVHD [91]. Participants received two doses (1–2 × 10^6^ cells/kg) with 100‐day primary assessment showing 86.7% overall response rate and 53.3% complete response rate, alongside 86.7% survival [91]. Critically, the 2‐year follow‐up confirmed sustained safety and efficacy: 60% survival (nine out of 15), no treatment‐related tumors or serious adverse events, and causes of death attributed to transplantation complications rather than cell therapy [257]. These outcomes are comparable to those natural MSC trials, establishing a critical proof‐of‐concept for iPSC therapeutics in inflammatory conditions.

However, further clinical translation requires continued optimization of reprogramming protocols, establishment of standardized good manufacturing practice‐compliant differentiation procedures, and comprehensive long‐term safety evaluation. Rigorous comparative studies between iPSC‐derived and naturally occurring stem cells will be essential to define the optimal therapeutic applications of this promising technology in disease management.

### 3.4 Cellular Product Refinement

**FIGURE 3:**
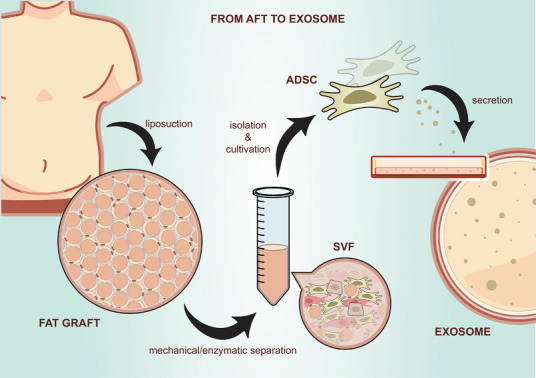
Therapeutic products derived from adipose tissue. Adipose tissue represents a hierarchical source of therapeutic products for the treatment of inflammatory diseases. The processing cascade begins with raw adipose tissue, which can be refined into stromal vascular fraction (SVF), from which adipose‐derived mesenchymal stem cells (ADSCs) can be isolated. Each stage of processing yields potentially therapeutic products, including cell‐free derivatives such as exosomes. The therapeutic efficacy of these adipose‐derived products is primarily attributed to their mesenchymal stem cell (MSC) characteristics, which confer immunomodulatory and regenerative properties.

As the understanding of stem cell therapy deepens, we have recognized that a range of upstream and downstream products may offer therapeutic benefits beyond traditional cellular preparations. This has led to a mindset shift toward developing minimally manipulated derivatives and acellular therapeutics, which strategically bypass many limitations of whole‐cell therapies. These refined products may harness native biological complexity while enhancing clinical practicality through structural preservation of regenerative niches, functional concentration of bioactive cargo, and logistical simplification for off‐the‐shelf deployment. Therefore, this section will systematically evaluate three innovative approaches: autologous fat transplantation (AFT), which exploits adipose tissue's innate regenerative capacity; stromal vascular fraction (SVF), which utilizes synergistic cellular interactions; and MSC‐derived exosomes, which deliver cell‐free therapeutic payloads (Figure 3).

#### 3.4.1 AFT

AFT, an established method in plastic and reconstructive surgery, involves tissue filling after liposuction. This minimally invasive technique offers a new surgical option for certain kinds of inflammatory conditions, such as FCD, SSc, and KOA.

The regenerative capacity of adipose grafts is primarily attributed to ADSCs within the adipose tissue matrix [258]. AFT's application in FCD treatment is based on several factors: (1) high ADSC content in grafts; (2) simplified procedure and reduced costs due to omission of isolation steps; (3) mechanical support and fistula filling function [259]; (4) enhanced ADSC proliferation under hypoxic conditions [260], potentially due to FGF‐2 release from injured adipocytes triggering JNK‐mediated proliferation and VEGF‐driven angiogenesis [261, 262]; (5) mature adipocytes’ ability to promote fibroblast recruitment [263], secrete regenerative lipocalin [264], and produce antimicrobial peptides [265]; (6) preadipocytes’ capacity to differentiate into mature adipocytes [266].

Clinical studies have shown promising results for AFT in treating recalcitrant rectovaginal fistulas [267] and anovaginal fistulas [268], with healing rates exceeding 80%. Optimized “doughnut technique” injections (circumferential grafting without tension) are critical for success [267]. A recent prospective interventional study reported an overall response rate of 76% in patients with complex perianal fistulas in CD [269], with outcomes comparable to those of purified ADSC therapy. A recent RCT involving 118 patients compared the therapeutic effects of AFT and PRP in KOA [270]. The results indicated that both treatments provided statistically and clinically significant improvements over 24 months, with comparably low rates of treatment failure and adverse events, and no disease progression was observed [270].

Alternative processing methods, such as the “Lipogems” technique for micro‐fragmented adipose tissue, have also shown encouraging results, achieving 67% single‐injection healing without enzymatic processing [271]. However, AFT faces limitations, primarily the variability in long‐term graft retention and the occurrence of necrotic areas posttransplantation [272, 273].

#### 3.4.2 SVF

SVF is a heterogeneous cell population containing fibroblasts, MSCs, smooth muscle cells, mural cells, macrophages, blood cells, and various stem cell phenotypes [274]. Isolation methods for SVF from adipose tissue vary, with mechanical and enzymatic separation being the primary approaches [275], though standardization remains challenging.

The diverse composition of SVF, while potentially beneficial, increases the risk of immune rejection, limiting its use primarily to autologous transplantation [276]. Despite this limitation, preliminary studies have shown promising results: (1) an observational study applying SVF instead of ADSCs on four patients suggested potential efficacy [277]; (2) the combination of SVF and autologous micro‐fat acts synergistically, with the microfat providing structural scaffolding and the SVF contributing bioactive signals [259]; (3) a case study of intravenous implantation of SVF in a psoriasis patient demonstrated a significant decrease in symptoms and reported no severe adverse events [278]; (4) a trial of local SVF injection for hand scleroderma reported no serious adverse events during surgery or follow‐up, with significant improvements observed in hand disability, pain, Raynaud's phenomenon, finger edema, and quality of life [279]. Sustained disease remission was documented during follow‐up extending up to 30 months [280, 281].

Comparative efficacy between MSCs and MSC‐derived products has also gained attention. A retrospective study comparing intra‐articular ADSCs versus SVF in KOA patients found that both contributed to clinical improvement, though ADSCs provided superior early symptom and pain relief with fewer comorbidities [282]. A prospective study echoed these results, showing that both ADSC and SVF injections significantly improved knee pain and function at all follow‐up time points, with ADSCs exhibiting significantly better outcomes in terms of rapid improvement of symptoms at median follow‐up [283]. Another recent retrospective study comparing SVF and AFT for KOA treatment reported improved VAS scores and mobility with both treatments; however, MRI observations indicated better cartilage preservation in less mechanically loaded areas and more durable therapeutic effects with AFT than with SVF [284].

However, despite the emergence of comparative efficacy studies, the specific therapeutic niches and optimal use cases for various ADSC‐derived products remain to be fully elucidated. Although most published studies have reported positive results [259, 267, 268, 269, 270, 271, 277, 278, 279, 283, 285, 286] (Table S1), several recent trials have failed to demonstrate superiority of ADSC‐based interventions over placebo [270, 285], cautioning against overgeneralization of efficacy and underscoring the need for deeper mechanistic insights and patient stratification.

#### 3.4.3 MSC‐Derived Exosomes

MSC‐derived exosomes, a cell‐free therapeutic modality, are currently considered a potential alternative to cell‐based MSC therapy, sparking a new wave of research in MSC‐based therapies. Many current studies investigating exosome applications have utilized MSC‐conditioned medium (MSC‐CM), which contains the complete milieu of MSC‐derived soluble factors and vesicular elements [287].

Several experimental studies have demonstrated the therapeutic potential of MSC‐derived exosomes: (1) Direct injection of MSC‐CM into mice with colitis showed therapeutic effects comparable to cell injection [288, 289]. (2) MSC‐exosomes exhibited similar immunomodulatory capabilities to intact MSCs [290]. (3) Like MSCs, MSC‐CM demonstrated responsiveness to preactivation. Preactivation of MSCs with proinflammatory factors significantly enhances their therapeutic capacity [291, 292]. (4) Topical application of MSC‐exosomes alleviates the chemical induced psoriasis‐like inflammation [293, 294]. (5) Exosomes can serve as drug carriers to target the joint cavity and improve collagen‐induced arthritis [295, 296]. (6) MSC‐derived exosomes can alleviate GVHD and preserve the graft‐versus‐leukemia effect in allogeneic stem cell transplantation animal models [297].

Several clinical trials have demonstrated the therapeutic potential of MSC‐derived exosomes in inflammatory disorders: (1) In plaque psoriasis, topical application of exosome‐HA sponges achieved 33% mPASI reduction and 41% plaque size decrease within 30 days, with parallel improvements in skin barrier function and elasticity [298]; (2) for cutaneous inflammation, intralesional injection of adipose‐derived exosomes significantly reduced psoriatic erythema/induration while elevating regulatory T‐cell marker FOXP3 and suppressing IL‐17/CD3+ T‐cell infiltration [299]; (3) in refractory FCD, localized exosome injections (5 mL/tract) induced complete fistula closure in 60% of patients and 69.7% of treated tracts, with histopathological confirmation of inflammation resolution and tissue regeneration [300]; (4) in pulmonary fibrosis, a phase I clinical trial involving 24 patients demonstrated that nebulized UCSC‐EVs combined with routine treatment were well tolerated with no serious adverse events, significantly improving lung function and respiratory health status [301]; (5) in primary Sjögren's syndrome‐related dry eye, a triple‐blinded randomized trial demonstrated that topical application of MSC‐derived exosome eye drops (10 µg twice daily for 2 weeks) significantly improved clinical outcomes (reduced ocular surface disease index scores, increased tear secretion, decreased fluorescein scores, and prolonged tear‐film break‐up time) compared with phosphate‐buffered saline controls [302].

These findings suggest that MSC‐derived exosomes may offer a promising cell‐free alternative to traditional MSC‐based therapies, potentially overcoming some of the limitations associated with cell transplantation while maintaining therapeutic efficacy. While the exosomes may not fully account for all MSC functions, they serve as a critical bridge between cellular and molecular therapeutics, with ongoing debates regarding cost‐benefit optimization for clinical translation.

Future research should focus on developing standardized protocols for these emerging therapeutic approaches, including optimizing AFT graft retention and SVF processing methods, as well as scaling up exosome production and enhancing organoid transplantation techniques. Comparative studies should complement this effort to determine the most effective treatment strategies for different disease manifestations.

## 4 Challenges and Limitations of Stem Cell Therapy for Inflammatory Diseases

### 4.1 Cell Preparation Procedure

Stem cell therapy faces unique challenges in cell sourcing and preparation compared with traditional medicines. The multistep process (harvesting, processing, and infusion) introduces significant variability. Autologous transplantation requires patients to undergo multiple procedures, hindering the widespread adoption of stem cell therapy [101, 183]. Allogeneic protocols also present challenges, including individual differences in cell sources, which lead to functional variations between cell products [303]. Moreover, processing variables compound these challenges, as cryopreservation methods (dimethyl sulfoxide vs. serum‐free media) significantly alter post‐thaw cell viability and paracrine function [304]. Variations in oxygen tension during expansion critically influence mitochondrial fitness and therapeutic potency [305]. The culture conditions like the choice of serum source [306] may introduce xenogenic contaminants or immune reactions, cumulatively resulting in significant efficacy variations that necessitate rigorous standardization through closed‐system bioreactors and validated potency assays for reliable clinical translation. This highlights the profound influence of manufacturing variables on cellular product quality and functional heterogeneity.

### 4.2 Limited Homing Ability and Longevity

The homing characteristics of MSCs remain controversial. Earlier studies have demonstrated MSCs' ability to migrate to sites of tissue damage and inflammation selectively [307]. Recent evidence implies that intravenously injected MSCs are primarily retained in the lungs, with rare instances of reaching target tissues [308, 309]. Intraperitoneal injection, often considered a form of systemic delivery for molecular drugs, presents unique conceptual and mechanistic uncertainties for cellular products. Critically, robust experimental evidence is lacking to confirm that cells are absorbed into the systemic circulation via the omental system, as is the case with small molecules. Within the complex peritoneal microenvironment, fundamental questions remain unresolved: which specific tissues or cells interact with administered stem cells, whether active homing capability is preserved or altered, how cell longevity is affected, and whether the promising therapeutic outcomes observed in animal models can be translated to humans.

Furthermore, whether intraperitoneal cell delivery essentially constitutes systemic administration or a localized approach remains undetermined [252, 310], which collectively questions the existence and magnitude of active homing. In the meantime, surviving MSCs face hostile microenvironments posttransplantation, including hypoxia, oxidative stress, and inflammation. These factors reduce engraftment time and cause continuous cell death [311, 312], resulting in only a small fraction of cells surviving for a limited period, thereby strongly limiting therapeutic longevity.

### 4.3 Safety and Side Effects

HSCT has been associated with significant mortality due to infections, GVHD, and various organ complications [313]. These high risks prevent HSCT from being a first‐line treatment for most of the inflammatory diseases. Notably, conditions such as IBD can also occur post‐HSCT [314, 315], suggesting potential transfer of genetic susceptibilities (e.g., NOD2/CARD15) from donor to recipient [314]. This raises questions about the need for genetic screening of donors to reduce the disease risks of recipients. Consequently, the inherent high‐risk profile of HSCT necessitates meticulous patient selection, presenting a significant clinical dilemma. Currently, its application is largely confined to complex, refractory cases or patients with comorbid hematological disorders or monogenic diseases. Nevertheless, even within this carefully selected cohort, the severity and incidence of adverse effects often remain substantially higher compared with alternative treatments. Therefore, while achieving an optimal risk–benefit balance is paramount, enhancing the intrinsic safety profile of HSCT remains an urgent and ongoing challenge.

MSC therapy generally has milder side effects, including allergies, hypothermia, and infections [97, 99, 102]. These effects are more common in systemic infusion regimens. While MSCs show no direct tumorigenic risk, their fusion with other cells may facilitate cancer metastasis [316]. Furthermore, amidst the rapid expansion of MSC therapeutics, heightened vigilance against unregulated proliferation is essential. The inherent complexity and lack of universal standardization across the cellular product lifecycle create vulnerabilities for both inadvertent and deliberate safety compromises. This underscores the critical importance of maintaining rigorous regulatory oversight, ensuring that only accredited medical institutions with appropriate expertise administer these therapies, and mandating the exclusive use of reliably sourced and rigorously characterized cellular products to safeguard patient safety.

### 4.4 Cost

The substantial financial burden associated with HSCT represents a significant barrier to its widespread adoption for inflammatory diseases. Treatment expenses exhibit considerable variability depending on the specific protocol employed. For allogeneic HSCT, total costs at the critical 100‐day mark can range dramatically from approximately $63,000 for reduced‐intensity conditioning regimens to an extraordinary $782,000 for complex procedures like double umbilical cord blood transplantation. Even at the 1‐year milestone, costs remain high, spanning from around $69,000 to over $637,000 [317]. While autologous HSCT generally presents a relatively lower cost profile compared with its allogeneic counterpart, it still imposes a heavy economic load, exemplified by an average total medical cost exceeding €66,000 for multiple sclerosis patients in Norway [318]. Collectively, these figures underscore that HSCT, regardless of the source, incurs exceptionally high expenditures. This profound economic burden fundamentally challenges the feasibility of implementing HSCT as a conventional, accessible therapeutic strategy for the broader population.

Cost data for MSC therapy remain less comprehensive and more fragmented than for HSCT. However, available information indicates a clear correlation: expenses escalate significantly with increasingly complex cell processing procedures and prolonged patient hospitalization periods. Consequently, a critical imperative emerges to simplify manufacturing workflows and rigorously standardize protocols across the board; such measures are indispensable prerequisites for meaningful cost containment. Intriguingly, preliminary analyses, such as the work by Dave et al., suggest a potential economic advantage for MSC therapy over established surgical interventions like fecal diversion in the specific context of fistula management [319], hinting at possible areas of cost‐effectiveness within the broader cell therapy landscape. Nevertheless, achieving broader economic viability for MSC therapies necessitates sustained efforts in process optimization and robust, transparent cost‐benefit analyses across diverse clinical applications.

### 4.5 Ethical Considerations

The accelerating advancement of cell therapy, driven by its transformative medical promise and substantial commercial appeal, has created a complex landscape of ethical dilemmas that extends far beyond conventional pharmaceutical development. Stem cell therapies, in particular, confront unique ethical challenges stemming from their biological complexity and regenerative nature. Unique challenges in stem cell therapy development include unclear definitions, unregulated authority, high processing complexity, elevated public expectations, and political sensitivities [320].

Fundamental issues include persistent ambiguities in defining critical parameters like cell identity, potency, and mechanism of action [321, 322], creating fertile ground for inconsistent product characterization and therapeutic claims. Across the full spectrum from preclinical research through manufacturing to clinical application, the absence of clearly delineated regulatory jurisdictions and harmonized oversight frameworks risks creating critical gaps in safety monitoring and accountability [323, 324]. The exceptionally intricate and variable nature of cell processing introduces profound ethical concerns regarding product consistency, quality control, and the potential introduction of undetected contaminants, directly impacting patient safety and therapeutic reliability. Compounding these technical challenges are often unrealistically high public and patient expectations, frequently amplified by premature media portrayals of “miracle cures,” which can erode informed consent processes and exploit vulnerable individuals desperately seeking relief. Furthermore, the field remains inherently intertwined with deep‐seated political sensitivities, particularly concerning the origins of specific cell types and the moral status attributed to biological materials [325, 326], which continue to shape research funding priorities and clinical accessibility in divergent ways.

As these multifaceted ethical, technical, and societal issues intensify alongside the field's expansion, a growing international consensus is emerging on the critical need for a robust, adaptive, and ethically grounded regulatory framework [327, 328, 329]. Failure to adequately address these ethical underpinnings risks not only individual harm but also significant setbacks for the entire field.

## 5 Strategies to Overcome Challenges and Optimize MSC Therapy

Several approaches have been explored to address current challenges and enhance the therapeutic efficacy of MSC therapy. iPSC technology, sharing similar properties with “natural” stem cells, may help resolve stem cell sourcing issues. Reliable systems now generate high‐purity definitive HSCs and MSCs from iPSCs [250, 330, 331]. iPSC‐derived cells exhibit comparable performance to natural stem cells in terms of cell proliferation, expansion capacity, immunomodulatory properties, and paracrine signaling effects. iPSCs serve as an effective source for tissue‐specific organoids production, particularly when patient biopsy data with rare gene mutations are unavailable [253].

Genetic modification of MSCs can enhance their inherent therapeutic properties by improving homing capabilities, enhancing survival rates, and boosting immunomodulatory effects [332]. Specific candidate molecules for modification have been selected for this purpose, including hypoxia‐response genes [333], antioxidant regulators [334], and adhesion molecules [335], which have shown promise in preclinical inflammatory models.

Efforts to improve the delivery and support of MSCs include (1) hydrogel encapsulation, which has demonstrated improved engraftment and enhanced immunomodulatory functions [336, 337]; (2) advanced delivery methods, where higher healing rates have been reported when stem cells are bound to fibrin glue and impregnated with fistula plugs [145], and (3) bioabsorbable scaffolds with concentrated MSCs show superior relatively high healing rates [338].

These strategies aim to address key challenges in MSC therapy, including cell sourcing, homing, survival, and delivery optimization. While these innovative approaches show promise, future research should focus on optimizing combination strategies that integrate iPSC‐derived MSCs with targeted genetic modifications and advanced delivery systems, as well as investigating the long‐term safety and efficacy of these enhanced therapeutic approaches through rigorous clinical trials across diverse patient populations.

Beyond technological advances, field‐wide progress necessitates robust nontechnical strategies, particularly the establishment of globally harmonized regulatory frameworks to ensure patient safety, product quality, and therapeutic validity. Ultimately, translating potential into clinical benefit requires synergistic integration of scientific innovation with stringent regulatory oversight, comprehensive standardization, and meticulous quality control.

## 6 Conclusion and Future Perspectives

Over the past decade, stem cell therapeutics have achieved remarkable progress in research and application, establishing multiple viable therapeutic paradigms. The clinical success of allogeneic HSCT in severe, treatment‐refractory cases has provided definitive proof‐of‐concept for cellular interventions in immune‐dysregulated disease subtypes. Concurrently, MSCs have demonstrated exceptional therapeutic versatility through both direct cell administration and minimally processed derivative products, including SVF and adipose tissue transplants. These approaches circumvent complex ex vivo expansion requirements while preserving critical cellular interactions and niche‐supporting factors. Cell‐free therapeutic modalities, particularly exosome‐based interventions, have demonstrated promising preclinical efficacy and are advancing toward clinical translation, providing new therapeutic avenues for treating refractory cases. Additionally, emerging research in TSCs and iPSC‐derived organoid systems is fundamentally reshaping our understanding of tissue regeneration mechanisms and advancing toward translational applications.

Despite these advances, significant translational barriers continue to impede the routine clinical implementation of stem cell therapies in inflammatory diseases. The multifactorial pathophysiology of these conditions creates a complex and dynamic therapeutic environment that frequently compromises treatment consistency and predictability. Many stem cell therapeutic modalities remain limited by suboptimal delivery methods, inadequate cell homing and engraftment, variable therapeutic responses, and a lack of comprehensive mechanistic understanding. Furthermore, several promising stem cell populations demonstrate primarily theoretical therapeutic potential, with limited robust preclinical validation to support clinical advancement.

To fully realize the transformative potential of stem cell therapeutics in inflammatory diseases management, the field must prioritize several critical research directions. First, comprehensive elucidations of multifactorial pathophysiological mechanisms for different inflammatory diseases will enable more precise therapeutic targeting and patient stratification strategies. Second, a systematic resolution of current therapeutic limitations is essential for clinical translation. This necessitates the optimization of cell delivery systems, the enhancement of engraftment efficiency, and the standardization of manufacturing protocols. Third, integration of cutting‐edge technologies, such as gene editing, advanced biomaterials, and personalized medicine approaches, will expand therapeutic possibilities and improve treatment precision. Ultimately, the accelerated translation of promising preclinical findings into rigorously designed clinical trials will be crucial for establishing the clinical utility and safety profiles across diverse inflammatory conditions.

The convergence of these research priorities, supported by sustained interdisciplinary collaboration and innovative clinical trial design, positions stem cell therapy to become a cornerstone of precision therapeutics for inflammatory diseases. By leveraging the unique regenerative and immunomodulatory capabilities of diverse stem cell populations, we can potentially transform treatment paradigms and significantly improve long‐term outcomes for patients with inflammatory diseases worldwide.

## Author Contributions

Chen Wu and Zhi‐Ping Jin performed the literature search and wrote the manuscript. Shu‐Qiang Weng prepared the figures and tables. Ji‐Min Zhu and Ling Dong supervised, reviewed, and revised the written manuscript. All authors have read and approved the final manuscript.

## Funding

This work was supported by the Shanghai 2022 “science and Technology Innovation Action Plan” Medical Innovation Research (Grant No. 22ZR1411800).

## Ethics Statement

The authors have nothing to report.

## Conflicts of Interest

The authors declare no conflicts of interest.

## Supporting information




**Supplementary Table 1**: Clinical trials using SVF/AFT for patients with inflammatory diseases.

## Data Availability

The data that support the findings of this study are available from the corresponding author upon reasonable request.
