# Supplementary material for: Stem Cell Therapy for Inflammatory Diseases: Progress, Challenges, and Future Directions
Source: MedComm (2020). 2026 Feb 2;7(2):e70616. doi: 10.1002/mco2.70616 (PMC12865230; doi:10.1002/mco2.70616)
Supplement: Supplementary file 1 — Supplementary Table 1: Clinical trials using SVF/AFT for patients with inflammatory diseases. [file MCO2-7-e70616-s001.docx]

**Stem cell** **therapy for inflammatory diseases: progress, challenges, and future directions**

Chen Wu^a^^, #^, Zhi-Ping Jin^b, #^, Shu-Qiang Weng^a^, Ji-Min Zhu^a,*^ and Ling Dong^a,*^

^a^*Department of Gastroenterology and Hepatology and Shanghai Institute of Liver Diseases,* *Zhongshan Hospital, Fudan University, Shanghai 200032, China.*

^b^*Department of Pharmacy, Zhongshan Hospital, Fudan University, Shanghai 200032,*

*China*

^*^Corresponding authors: [zhu.jimin@zs-hospital.sh.cn](mailto:zhu.jimin@zs-hospital.sh.cn) (JMZ) and [dong.ling@zs-hospital.sh.cn](mailto:dong.ling@zs-hospital.sh.cn) (LD)

^#^These authors contribute equally to this work.

Supplementary Table 1: Clinical trials using SVF/AFT for patients with inflammatory diseases

| **Year** | **Disease** | **Phases** | **patients** | **Methods** | **Follow up (month)** | **Efficacy** | **Adverse event** | **NCT No. (status)** | **Ref** |
| --- | --- | --- | --- | --- | --- | --- | --- | --- | --- |
| 2009 | FCD | NA | 4 | SVF | 12 | 25% fistula healing rate | no AE/SAE | NA | ^277^ |
| 2015 | FCD | NA | 2 | AFT | 23-25 | 0 fistula healing rate (healed at the first 6-12w) | NA | NA | ^267^ |
| 2018 | FCD | NA | 7 | AFT | 25 | 86% fistula healing rate | NA | NA | ^268^ |
| 2019 | FCD | NA | 21 | AFT | 6 | 57% fistula healing rate | 4 proctalgia, 2 abscess,1 bleeding, 1 urinary retention | NCT03803917 (Completed) | ^269^ |
| 2019 | FCD | I/II | 10 | SVF + AFT | 12 | 60% fistula healing rate | 40% moderate pain due to liposuction, 3 SAE | NCT02520843 (Completed) | ^259^ |
| 2020 | FCD | NA | 15 | AFT | 6 | 66.7% (combined remission); 93.3% (clinical remission) fistula healing rate | 20% subcutaneous hematoma | NCT03555773 (Completed) | ^271^ |
| 2015 | SSc | NA | 12 | SVF | 6 | significant improvements in symptoms | 4 spontaneously resolved minor AEs | NCT01813279 (Completed) | ^279^ |
| 2022 | SSc | II | 40 | SVF | 3 | improvement of hand function in both groups, no superiority in SVF group | 35 AE; 8 related SAE | NCT02558543 (Terminated) | ^285^ |
| 2018 | psoriasis | NA | 1 | SVF | 12 | significant decrease in symptoms with a noticeable difference in skin quality appearance | no AE/SAE | NA | ^278^ |
| 2019 | KOA | NA | 16 | SVF | 12 | significantly improvement in the VAS, WOMAC scores, and ROM | 4 abdomen pain (liposuction related); 6 joint pain and swelling (injection related); no SAE | NA | ^286^ |
| 2022 | KOA | NA | 118 | AFT | 24 | statistically and clinically significant improvement, not superior to platelet-rich plasma | NA | NA | ^270^ |
| 2022 | KOA | NA | 80 | SVF/ADSC | 24 | ADSC and SVF injections both substantially improved knee pain and function; ADSC take effect quicker | joint pain and swelling | NA | ^283^ |

SVF, Stromal vascular fraction

AFT, Autologous fat transplantation
